# The Diversity of REcent and Ancient huMan (DREAM): A New Microarray for Genetic Anthropology and Genealogy, Forensics, and Personalized Medicine

**DOI:** 10.1093/gbe/evx237

**Published:** 2017-11-20

**Authors:** Eran Elhaik, Leeban Yusuf, Ainan I J Anderson, Mehdi Pirooznia, Dimitrios Arnellos, Gregory Vilshansky, Gunes Ercal, Yontao Lu, Teresa Webster, Michael L Baird, Umberto Esposito

**Affiliations:** Department of Animal and Plant Sciences, University of Sheffield, United Kingdom; Independent researcher, Newick, United Kingdom; Department of Psychiatry and Behavioral Sciences, Johns Hopkins University; Department of Biology, Lund University, Sweden; National Geographic Society, Washington, District of Columbia; Department of Computer Science, Southern Illinois University Edwardsville; Thermo Fisher Scientific, Santa Clara, California; DNA Diagnostics Center, Fairfield, Ohio

**Keywords:** population genetics, biogeography, ancient DNA, archaic DNA, forensics, CNVs

## Abstract

The human population displays wide variety in demographic history, ancestry, content of DNA derived from hominins or ancient populations, adaptation, traits, copy number variation, drug response, and more. These polymorphisms are of broad interest to population geneticists, forensics investigators, and medical professionals. Historically, much of that knowledge was gained from population survey projects. Although many commercial arrays exist for genome-wide single-nucleotide polymorphism genotyping, their design specifications are limited and they do not allow a full exploration of biodiversity. We thereby aimed to design the Diversity of REcent and Ancient huMan (DREAM)—an all-inclusive microarray that would allow both identification of known associations and exploration of standing questions in genetic anthropology, forensics, and personalized medicine. DREAM includes probes to interrogate ancestry informative markers obtained from over 450 human populations, over 200 ancient genomes, and 10 archaic hominins. DREAM can identify 94% and 61% of all known Y and mitochondrial haplogroups, respectively, and was vetted to avoid interrogation of clinically relevant markers. To demonstrate its capabilities, we compared its *F*_ST_ distributions with those of the 1000 Genomes Project and commercial arrays. Although all arrays yielded similarly shaped (inverse J) *F*_ST_ distributions, DREAM’s autosomal and X-chromosomal distributions had the highest mean *F*_ST_, attesting to its ability to discern subpopulations. DREAM performances are further illustrated in biogeographical, identical by descent, and copy number variation analyses. In summary, with approximately 800,000 markers spanning nearly 2,000 genes, DREAM is a useful tool for genetic anthropology, forensic, and personalized medicine studies.

## Background

The field of population genetics experienced astonishing changes over the past 50 years, generating a new understanding of variability at the molecular level that has allowed for the exploration of new biological paradigms. Over the past decade, this turmoil was driven by the wide availability of single-nucleotide polymorphism (SNP) microarray and next generation sequencing (NGS) data, which raised major questions concerning human early origins, interbreeding with archaic hominins, and the processes that shaped inter- and intrapopulation variability.

Such questions are also the core components of forensic DNA phenotyping. Common forensic and mass disaster scenarios alongside accumulated criticism of eyewitness testimonies necessitated the development of more accurate and reliable DNA-based forensics tools based on short tandem repeats (STRs) extracted from minute DNA amounts (Kayser 2015). The a growing demand for accurate profile reconstructions from DNA evidence, beyond STR, dubbed “DNA intelligence,” led to the development of the Forensics Chip and “calculators” for skin and eye colors (Kayser 2015), yet an updated microarray that incorporates recently found forensic markers does not exist.

Interest in human ancestry is not limited to genetic anthropologists, genealogists, and forensic experts. The relatedness of adaptations to diseases is instrumental to identify targets for drug treatment (Sheridan 2015). The appreciation that demographic histories, geographical origins, and migration patterns shaped the genetic risk to disorders and treatment response (Yusuf and Wittes 2016) underlies personalized medicine. This allows purporters of personalized medicine vouch for a more comprehensive molecular information on patients through genomics and other “omics” data.

Since NGS technologies remain prohibitively expensive, microarray SNP technology became the “workhorse” for geneticists, although they are limited in a number of ways. First, genotyping data are susceptible to ascertainment bias due to the choice of SNPs (Albrechtsen et al. 2010). Although there has been an increase in the numbers of genotyped indigenous populations, estimated at 5,000–6,000 groups (Fardon 2012), commercial microarrays still rely on the four HapMap populations (illumina 2010). More recent arrays use some or all the 26 1000 Genomes Project (GP) populations (Thermo Fisher Scientific), but representing the complete human biodiversity continues to be a challenge. This has several negative effects in limiting the phylogeographic resolution of the findings and maintaining health disparities (Popejoy and Fullerton 2016). Second, microarray content is typically reflective of data known or considered at the time of the design of the array. Finally, most microarrays were not designed to allow inference of copy number variations (CNVs), which are useful in studying various phenotypes and depicting population structure.

Motivated by progress in the studies of modern and ancient genetic diversity, adaptation mechanics, forensic phenotypes, and drug response, we aimed to design an affordable and all-inclusive microarray. Our goals were to: 1) design The Diversity of REcent and Ancient huMan (DREAM)—a state of the art SNP microarray dedicated to genetic anthropology and genealogy, forensics, and personalized medicine; 2) validate its accuracy; 3) evaluate its abilities to discern populations compared with alternative arrays; and 4) assess its performances on worldwide populations.

## Materials and Methods

### Genetic Data Retrieval

Ancestry informative markers (AIMs) were obtained from the same 15 studies as listed in Elhaik et al. (2013). Anonymous genotype data of 606 unrelated individuals from 57 populations genotyped on the GenoChip microarray as part of the Genographic Project and their sampling sites were obtained from Elhaik et al. (2014).

Ancient DNA genomic data were obtained from 11 publications depicting 207 ancient genomes (Keller et al. 2012; Raghavan et al. 2014; Fu et al. 2014; Gamba et al. 2014; Lazaridis et al. 2014; Olalde et al. 2014; Seguin-Orlando et al. 2014; Skoglund et al. 2014; Allentoft et al. 2015; Haak et al. 2015; Llorente et al. 2015; Schiffels et al. 2016). In the case of sequence data, sequence reads were aligned to the human reference assembly (UCSC hg19—http://genome.ucsc.edu/; cited 2017 Jul 16) using the Burrows Wheeler Aligner (BWA version 0.7.15) (Li and Durbin 2009), allowing two mismatches in the 30-base seed. Alignments were then imported to binary (bam) format, sorted, and indexed using SAMtools (version 1.3.1) (Li et al. 2009). Picard (version 2.1.1) (http://broadinstitute.github.io/picard/; cited 2017 Jul 16) was then used for MarkDuplicates to remove reads with identical outer mapping coordinates (which are likely PCR artifacts). The Genome Analysis Toolkit RealignerTargetCreator module (GATK version 3.6) (McKenna et al. 2010; DePristo et al. 2011) was used to generate SNP and small InDel calls for the data within the targeted regions only. GATK InDelRealigner/BaseRecalibrator was then used for local read realignment around known InDels and for base quality score recalibration of predicted variant sites based on dbSNP 138 and 1000 Genomes known sites, resulting in corrections for base reported quality. The recalibration was followed by SNP/InDel calling with the GATK HaplotypeCaller. Variants were filtered for a minimum confidence score of 30 and minimum mapping quality of 40. At the genotype level, all genotypes that had a genotype depth <4 or a genotype quality score <10 were removed from the data set by setting them to missing in the VCF. GATK DepthofCoverage was then used to re-examine coverage following the realignment. VCFtools (version 0.1.14) (Danecek et al. 2011) were used to convert the VCF file to PLINK format (Purcell et al. 2007). We used Haak et al.’s (2015) chronology. We obtained the low- and high-coverage sequences data for Neanderthal (Green et al. 2010) and Denisovan genomes (Meyer et al. 2012; Sawyer et al. 2015).

### SNP and Haplogroup Validation 

To cross-validate DREAM’s genotypes, we genotyped 139 individuals from 17 worldwide 1000 GP populations including: Americans of Mexican ancestry (Los Angeles), Bengali (Bangladesh), British (England and Scotland), Caribbean Africans (Barbados), Colombians (Medellin, Colombia), Esan (Nigeria), Finnish (Finland), Gambian (Western Division, The Gambia), Han Chinese (Beijing, China), Iberian (Spain), Indian Telugu (UK), Italians (Tuscany, Italy), Kinh (Ho Chi Minh City, Vietnam), Mende (Sierra Leone), Peruvians (Lima, Peru), Punjabi (Lahore, Pakistan), and Yoruba (Ibadan, Nigeria). Genotypes were produced following the Axiom Best Practices Genotyping Analysis Workflow (Thermo Fisher Scientific 2017), which executes sample and marker QC. The concordance between DREAM and 1000 GP (phase 1) genotypes was calculated as the proportion of the genotypes (AA, AB, and BB) that were identical between the two data sets. The marker call rate was calculated as the proportion of genotypes that were not set to No Calls out of the total genotype calls attempted.

Maternal and Paternal haplogroup calling was done using an internal haplogroup calling algorithm developed by the Genographic Project, as in Elhaik et al. (2013).

### Comparing Summary Statistics between Genotyping Arrays

DREAM’s autosomal and X-chromosomal SNPs ability to differentiate populations was compared against alternative platforms. For each platform, we calculated the alternative allele frequency (AF) and *F*_ST_ based on 1000 GP phase 3 data (Durbin et al. 2010) provided by the Ensembl Variant Effect Predictor (McLaren et al. 2016). Calculations were based on unrelated Europeans (CEU), Africans (YRI), and Han Chinese (CHB). Aside DREAM, the compared platforms include the complete 1000 GP data set (87,829,960 SNPs), a reduced subset of 1000 GP without rare SNPs (MAF < 0.01) (14,426,697 SNPs), and four microarrays: HumanOmni5 (illumina 2015) (4,156,080 SNPs), HumanOmni2.5 (illumina 2013) (2,226,048 SNPs), Infinium Multi-Ethnic Global (illumina 2016) (1,486,126 SNPs), and Human Origins (Lu et al. 2011) (627,981 SNPs).

Due to the large number of *F*_ST_ values in each data set, their length distributions are very noisy. We thus adopted a simple smoothing approach in which *F*_ST_ values are sorted and divided into 1,000 equally sized subsets. The distribution of the mean *F*_ST_ value is then calculated using a histogram with 40 equally sized bins ranging from 0 to 1. To test whether two such *F*_ST_ distributions obtained by different arrays are different, we applied the Kolmogorov–Smirnov goodness-of-fit test and the false discovery rate (FDR) correction for multiple tests (Benjamini and Hochberg 1995). Because the differences between the distributions were highly significant due to the large sample sizes, we also calculated the effect size, first by using the nonoverlapping percentage of the two distributions, and then by using Hedges’ *g* estimator of Cohen’s *d* (Hedges 1981). If the area overlap is >98% and Cohen’s *d* is <0.05, we considered the magnitude of the difference between the two distributions to be too small to be biologically meaningful.

Next, we compared the identical by descent (IBD) coverage obtained by each microarray. IBD varies by individual, population, proportion of rare alleles, and number of SNPs. For that, we assembled an autosomal data set by randomly selecting 30 individuals from three 1000 GP populations (phase 3) that have the same proportion of rare alleles (MAF < 0.5%) (Genomes Project et al. 2015). Analyses were carried out using only the autosomal SNPs of each microarray. For each individual, we retained the average IBD with all individual of the same population. We then calculated the mean and standard deviation per population and divided them by the number of SNPs of the microarray.

Finally, we compared the linkage disequilibrium (LD) patterns between the microarrays. For that, we used the 1000 GP (phase 3) data set. We randomly selected 30 individuals from four populations: Yoruba (Ibadan, Nigeria), Finnish (Finland), Japanese (Tokyo, Japan), and Puerto Ricans (Puerto Rico). We then analyzed the SNPs sequenced in those populations that were included in each of the five genotyping arrays: DREAM (688,320) HumanOmni5 (3,845,760 SNPs), HumanOmni2.5 (2,155,999 SNPs), Infinium Multi-Ethnic Global (1,319,453 SNPs), and Human Origins (564,019 SNPs). Lastly, we calculated the LD statistic (*r^2^*) using the PLINK (Purcell et al. 2007) command: –ld-window-r2 0 –r2 –ld-window 2, which calculated *r^2^* for each variant pair with two variants between them at most (ld-window = 2) without filtering low *r^2^* values (window-r2 = 0).

### Identical by Descent Analysis with Archaic Hominins

We assembled an autosomal data set by randomly selecting 30 individuals from 15 1000 GP populations (phase 3). Using BCFtools (Li 2011), we merged this data set with genotypes of the Denisovan, Neanderthal, and the chimpanzee reference genome panTro4 (Feb. 2011) obtained from the UCSC genome browser. We filtered low-quality positions (marked as LowQual), InDels, and uncalled genotypes with VCFtools (version 0.1.14) (Danecek et al. 2011). We also removed positions that did not differ from the reference allele for all samples using “–non-ref-ac-any 1.” The final data set contained 36,375,129 SNPs. A subset of this data set contained 669,954 autosomal DREAM SNPs.

We applied Refined IBD implemented in Beagle version 4.1 (21Jan17.6 cc) with the reference human genome (Browning and Browning 2013) to both data sets. To improve the small segments detection ability, we used ibdtrim = 0 and ibdcm = 0.001.

### Biogeographical Origins of Worldwide Individuals

Biogeographical predictions were obtained with the geographical population structure (GPS) following Elhaik et al. (2014). GPS accepts the DNA of an unmixed individual and estimates its admixture components in respect to nine admixture components corresponding to putative ancestral populations. It then matches the admixture proportions of the individual to those of *reference populations* known to have resided in a certain geographical region for a substantial period of time. GPS then converts the genetic distances between that individual and the nearest *M *=* *10 reference populations into geographic distances. The *reference populations* can be thought of as “pulling” the individual toward their location in a strength proportional to the similarity of their admixture components until a “consensus” is reached (Das et al. 2017).

DREAM’s biogeographical ability was assessed using the Genographic data set. The 23,782 autosomal SNPs overlapped between the GenoChip and DREAM were used to infer nine admixture components (supplementary fig. S1, Supplementary Material online), which were provided as input for GPS. Individuals were grouped into their populations. Subpopulations were computationally determined by employing MATLAB’s *k*-means clustering and multiple pairwise *F* tests on populations with *N_p_*>4, where *N_p_* is the number of individuals within a population. For *k *=* *2 to *k *=* N_p_*/2, we used *k*-means to identify *k* clusters and then the ANOVA *F* test to test whether cluster pairs are significantly different (*P *<* *0.05). If the hypothesis is verified for all the pairs at iteration *i*, then another iteration follows until at least one pair violates the hypothesis and *k_i-1_* is the optimal number of clusters. Populations and subpopulations displaying only one individual were discarded from the data set. The final data set consisted of 587 individuals grouped into 123 subpopulations from 33 countries (supplementary tables S8 and S9, Supplementary Material online). These subpopulations were considered *reference populations*. The admixture components of the *reference populations* were determined by their average.

We localized the 584 individuals using the full *reference population* data set, the leave-one-out individual, and leave-one-out subpopulation approaches. Two measures were used to assess the biolocalization accuracy: first, a binary index indicated whether an individual is predicted within 200 km from the border of their true country. Second, the distance between the predicted and true location was calculated with the Haversine formula.

### Genetic Similarity between the Worldwide Individuals

To calculate the genetic similarity between individuals, we first created a minimum connectivity *k*-nearest neighbors (k-NN) graph *G* based on the 9 admixture components (supplementary fig. S1, Supplementary Material online). We then clustered *G* by applying the novel graph-theoretic node-based resilience clustering framework NBR-Clust (Matta et al. 2016). The various node-based resilience measures, such as vertex attack tolerance, integrity, tenacity, and toughness, compute a relatively sparse critical attack set of nodes, whose removal causes severe disruption to the network connectivity, outputting the result of an optimization function representing the difficulty of disrupting the network as a specific measure of the network resilience. NBR-Clust takes any node-based resilience measure *r* as a parameter and performs noise-robust clustering on *G* primarily by outputting the connected components resulting from the removal of the critical attack set computed by *r*(*G*) as the basic clusters. If noise or overlap exists, outlier nodes are computed as a subset of the critical attack nodes, which form the cluster boundaries in *G.* We used *integrity* as the node-based resilience measure to cluster *G* due to its noted robustness when the number of ground truth clusters is not known *a priori*. Our integrity-based graph clustering results in eight clusters, each corresponding to a different color in the figure. The graphs are visualized using the Gephi 0.9.1 graph visualization program (Bastian et al. 2009).

The different sizes of the nodes (and node labels) were created using the *betweenness centralities* property BC(v): BC(*v*) of a node *v* is the sum over all pairs of other nodes *x, y*, of the ratio of the number of *x–y* shortest paths that go through node *v* to the total number of x–y shortest paths. As BC(*v*) measures the extent to which *v* lies between other nodes (as well as between multiple clusters), larger nodes are intermediate to more pairs of graph nodes than smaller nodes in the visualization. Thus, the highest betweenness, larger nodes tend to lie on the boundaries between clusters in the NBR-Clust framework, representing outliers in terms of cluster overlap or noise. As such, we hypothesize that the largest, that is, highest betweenness centrality, nodes represent individuals with higher levels of admixture with respect to the clusters to which they are adjacent.

### CNV Analysis

To infer CNVs, we applied the Axiom CNV Summary Tool (Thermo Fisher Scientific 2015) to the 139 1000 GP individuals genotyped in DREAM. The tool uses signal intensity and genotypes to calculate log2 ratios and B allele frequencies (BAFs) from normalized probeset signal data. Since the CNVs inferred for the 1000 GP individuals cannot be directly validated, we aimed to replicate the population structure patterns reported by Sudmant et al. (2015).

A CNV was considered valid if a change in the signal intensity was identified in at least 40% of the markers that covered it. To reduce biases in PCA, we selected 11 random individuals from Africa, America, Europe, and East Asia. Since many of the CNVs were not included in DREAM due to their ability to discern populations, we narrowed our analyses to CNVs covered by at least 15 markers that were unique to one regional population and to individuals that harbored at least 15 CNVs. We carried out a PCA analysis on the remaining 132 deletions and 97 duplications. The *PlotGenome* script (Elhaik and Graur 2013) was used to draw the chromosomal view.

## Results and Discussion

### Designing the DREAM SNP Microarray

The DREAM microarray (Axiom_DDCGPS01) was designed as an Applied Biosystems Axiom custom array. The Axiom genotyping platform utilizes a two-color ligation-based assay using 30-mer Oligonucleotide probes synthesized in situ onto a microarray substrate. There are ∼1.38 million features (or cells) with each SNP feature containing a unique 30mer oligonucleotide sequence complementary to the sequence flanking the polymorphic site on either the forward or the reverse strand. Depending on the 3′ (SNP-site) base (A or T, vs. C or G), solution probes bearing attachment sites for one of two dyes are hybridized to the target complex, followed by ligation for specificity. DREAM was designed with 809,781 oligonucleotide sequences complementary to the forward or reverse strands (probesets) that interrogate 799,120 markers (SNPs or InDels). The following sections detail how markers were selected to enable ancestry, genealogy, forensics, and personalized medicine applications.

#### Ancestry Informative Markers

**Fig. 1.**
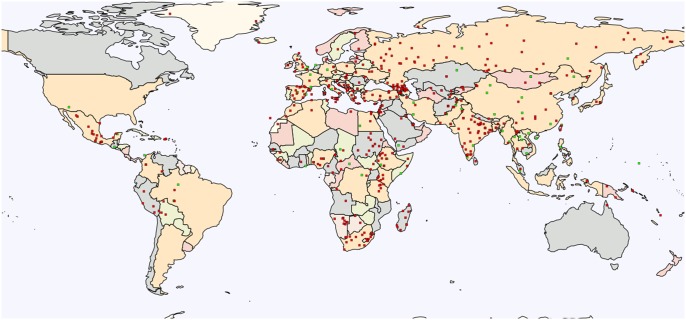
—Worldwide distribution of population from which AIMs were obtained. AIMs from over 450 world populations were harvested from the literature (green) or calculated based on genotyped data from public collections (red).

AIMs are invaluable tools in population genetics and genetic anthropology as they allow the identification of populations that vary in substructure, quantification of the degree of admixture, and detection of subtle population subdivisions using a limited number of markers (Enoch et al. 2006). We collected 50,504 AIMs (49,555 autosomal and 949 X-chromosomal): one-third (15,591) were culled from the literature that encompassed over 450 populations (fig. 1). The remaining AIMs were selected randomly and uniformly from the GenoChip’s autosomal, and X chromosomal AIMs that were obtained from over 300 populations (Elhaik et al. 2013).

#### Ancient DNA Markers

Ancient DNA from sequence or genotype data allows direct observations of past admixture and migration events and is often the only evidence that allows the examination of historical hypotheses. As such, ancient DNA studies have provided insights into human evolution and migration (Morozova et al. 2016). We curated genetic data from over 200 ancient genomes (supplementary table S1, Supplementary Material online). Due to the data sparsity, we strived to select markers shared across as many genomes as possible to minimize the overall number of SNPs while retaining sufficient data (approximated at 1,000 SNPs) from each genome. For that, a greedy algorithm applied to all the genomes iteratively selected the SNPs with the maximal number of alleles available for most of the genomes. Each SNP at a time was marked for inclusion, omitted from the data set, and the process of SNP selection repeated until each genome was sufficiently covered by at least 1,000 SNPs. SNPs from genomes consisting of only a few hundred SNPs were manually added to provide effective coverage.

To facilitate studies on the extent of gene flow from Neanderthal and Denisovan populations to modern humans, we included SNPs from multiple low-coverage genomes while restricting the selection to markers validated by the 1000 GP. As such, we randomly selected 1,000 and 3,000 SNPs for Denisovans 4 and 8, respectively, and 5,000 SNPs from six Neanderthals (Feld1, Mezmaiskaya, Sid1253, Vi33_25, and Vi33_26). Overall, we selected 78,724 markers (73,107 autosomal and 5,617 X-chromosomal), 12,550 of which were culled from archaic hominin genomes.

#### Adaptation Markers

**Fig. 2.**
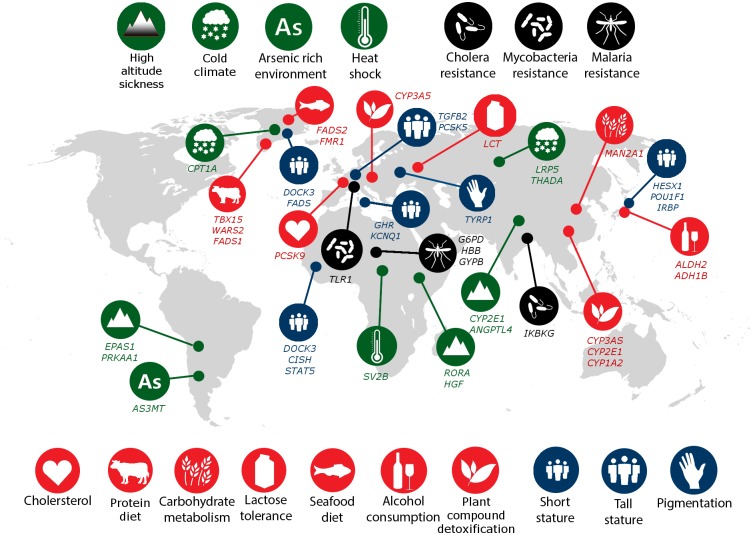
—Local human adaptations. Following Fan et al. (2016), each adaptation is labeled by the phenotype and/or selection pressure. The genetic loci under selection and the studied population are shown.

Adaptive responses to selective pressures in particular geographic regions have become increasingly important in understanding human history (Jobling et al. 2013; Racimo et al. 2017). Populations experiencing selective pressures were instrumental in identifying the genetic variants that confer these adaptive qualities. For example, the modulation fatty-acids and growth hormone in Greenland Inuits was found to be influenced by two markers located in *FADS1* and *FADS2* (Fumagalli et al. 2015). Following previous mapping efforts and based on the literature published over the past 5 years, we constructed a comprehensive list of adaptive traits and curated variants and genes that are significantly associated with those traits. Genes significantly associated with adaptations of interest were recorded and included in the design (fig. 2 and supplementary table S2, Supplementary Material online).

#### Forensic Informative Markers and Other Traits

**Fig. 3.**
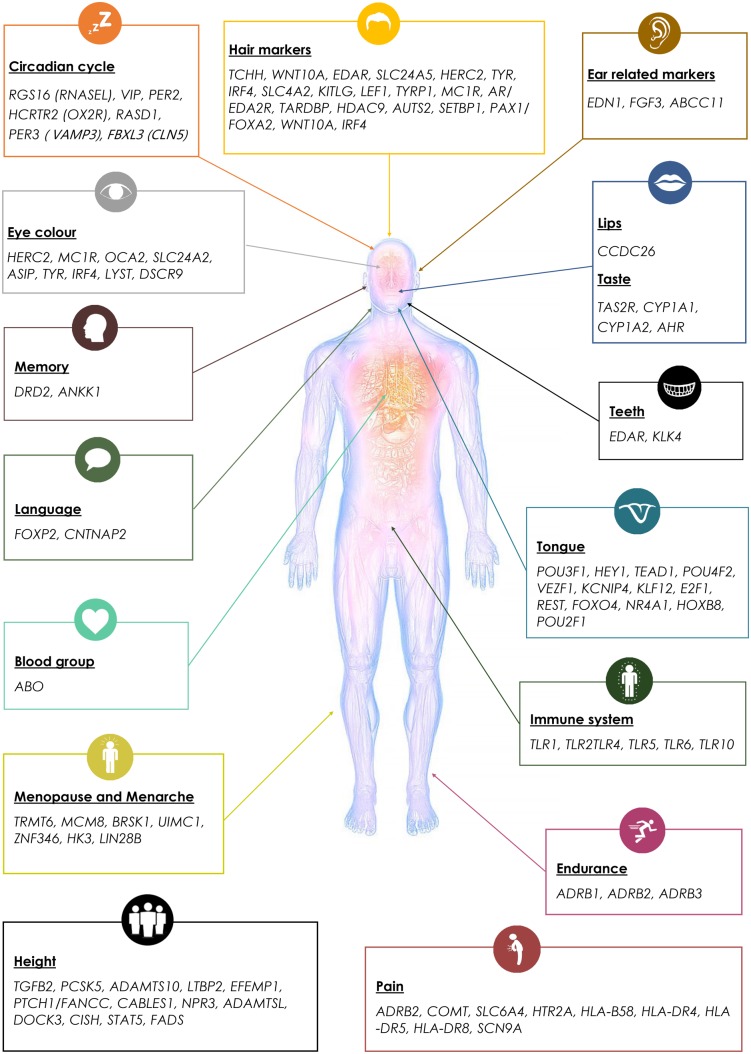
—Human traits and their associated genetic loci.

To facilitate forensic studies, we aimed to infer forensic informative markers (FIMs) for DNA phenotyping. Following previous studies (Kayser 2015) and based on academic publications made over the past 5 years, we developed a panel of forensic-relevant traits and curated FIMs and genes that are significantly associated with those traits. We also included in the design markers and genes associated with popular traits such as memory, language, circadian cycle, immune system, and endurance (fig. 3 and supplementary table S3, Supplementary Material online).

#### Enabling CNV Analyses

CNVs have contributed significantly to hominid evolution (Sudmant et al. 2013), biodiversity (Freeman et al. 2006), adaptations, traits, and disease (Sudmant et al. 2015; Zarrei et al. 2015). CNVs may also be useful tools in forensics, similar to that played by STRs. The ability to detect SNPs and CNVs in the same genome screen is thereby advantageous to genetic anthropology, forensics, and epidemiology.

Applied Biosystems Axiom arrays from Thermo Fisher Scientific can be designed to detect both SNPs and CNVs. Applied to whole-genome data from a set of human cell lines with large chromosomal aberrations, Webster et al. (2013) showed that in regions with sufficient probe density, both copy number gains and losses can be detected with high overall sensitivity and high breakpoint accuracy. We selected 351 genomic regions of varying lengths ($\overset{-}{L}$=125,452; $\tilde{L}$= 23,647 bp) that were sufficiently large (*L *>* *10,000 bp) or shown to differentiate populations (Sudmant et al. 2015). These regions were covered by 29,195 probesets, designed by Thermo Fisher Scientific at an average spacing of ∼1,500 bp. A majority of the regions (306) were covered by 25 probesets or more to ensure detection accuracy (supplementary fig. S2 and table S4, Supplementary Material online).

#### Personalized Medicine Markers

To enable precision medicine applications, we selected pharmacogenetic SNPs from public repositories and the literature. SNPs were culled from the Pharmacogenomics Knowledgebase (PharmGKB), whose data are associated with human genetic variation in drug responses (Whirl-Carrillo et al. 2012) (∼75% of 3,476 SNPs annotated by PharmGKB were collected), and from the Applied Biosystems Drug Metabolizing Enzymes and Transporters DMET) microarray (Sissung et al. 2010), whose genes are related to drug absorption, distribution, metabolism (∼60% of 1,924 SNPs were collected). Genes and SNPs implied by the eMERGE network to be associated with phenotypic outcome like pain (e.g., *SCN10A*), Hypothyroidism (e.g., *FOXE1*), cholesterol (e.g., *CETP* and *LIPC*), platelets, and red and white blood cells (Crawford et al. 2014) were also included. We further included SNPs and genes associated with Warfarin response like *VKORC1*, *CYP2C9*, *ADRB1*, *ADRA2C*, and *BEST* (Johnson 2008; Scott et al. 2008; Daneshjou et al. 2014) and nearly all the cytochrome P450 genes associated with drug metabolism. Lastly, we included genes associated with aging (Shadyab and LaCroix 2015).

#### All Other Genome-Wide Markers

Studies of sex bias in human admixture, migrations, and kinship analyses typically require a high coverage of the X chromosome. We thereby enriched the X chromosomes with SNPs selected uniformly throughout the genome. We prioritized SNPs that had Applied Biosystems Axiom confirmed probes and those that are targeted by Illumina’s HumanOmni5 array. Overall, 50,265 SNPs were selected.

Of particular importance is the major histocompatibility complex (MHC) locus involved in autoimmune and infectious diseases. The MHC region is the most gene-dense region in the human genome. However, the high density in polymorphisms and linkage disequilibrium have limited our understanding of its role. To facilitate further research of this locus, we included SNPs for which Applied Biosystems Axiom had confirmed probesets and that reside within the 4 M bp of the MHC. Overall 16,434 SNPs were selected.

To enable further research into traits of interests, we targeted SNPs that reside within or in the 100 kilobases flanking regions of the genes of interest. We used STRING (Jensen et al. 2009) to find genes associated with the genes of interest (figs. 2 and3). In some cases, the entire gene families of genes (e.g., keratin and cytochrome P450) strongly associated with the phenotypes of interest were included in the design.

To enable cross-platform kinship analyses, we selected ∼230,000 SNPs distributed uniformly throughout the genome that had Applied Biosystems Axiom confirmed probesets.

Finally, we interrogating over nearly 14,000 markers to identify SNPs defining Y and mtDNA haplogroups (supplementary material S1, Supplementary Material online).

#### Vetting the Array

We excluded most of the SNPs that required four probesets or more unless they were vital to call haplogroups. To improve coverage, we prioritized SNPs that required a single probset over those that required two. We also filtered out all the markers that were recorded in the ClinVar database (Landrum et al. 2016) (as of 2/23/2016). We thus designed a multipurpose genotyping array dedicated for genetic anthropology and genealogy, forensics, and personalized medicine.

### Validating the DREAM Microarray Results

After excluding unreliable Y and mitochondrial markers, the final DREAM microarray targets 794,302 markers: 730,581 autosomal and pseudoautosomal, 48,973 nonpseudoautosomal (nonPAR) X, 13,576 Y-chromosomal, and 1,172 mitochondrial markers without clinical relevance. The design spans over 1,903 genes (supplementary table S5, Supplementary Material online) enriched with members of the collagen (46), keratin (155), cytochrome P450 (68), forkhead box (FOX) (22), RNA polymerase subunits (POLR) (34), solute carrier (38), and interleukin (22) gene families. Of DREAM’s autosomal, nonPAR X, Y and mtDNA SNPs, 95.8%, 98.6%, 57.0%, 73.3%, respectively are found in the 1000 GP (phase 3). Coincidently, DREAM also shares a significant number of SNPs with other commercial arrays, but never more than 40% with any array.

Genotype accuracy was assessed by genotyping 139 individuals from 17 worldwide populations found in the 1000 GP data (Altshuler et al. 2010) and cross-validating them with the 1000 GP data. About 100% (139/139) of the samples passed sample QC, and 97.5% (774,648/794,302) of the markers passed marker QC. For autosomes that passed marker QC, the concordance rate was 99.70% (88,753,010 genotypes agree/89,019,543 total genotypes) and the total marker call rate was 99.70% (102,164,485AA + AB + BB genotypes/102,468,039 AA + AB + BB+ No calls). For the nonPAR X markers, the concordance between the genotypes from the 46,020 markers (included, passed markerQC, and part of the 1000 GP phase 3) and the 1000 GP phase 3 genotypes was calculated as 99.76% (5,955,934/5,970,139). For the Y chromosome, the concordance between the genotypes from the 7,745 markers (part of the 1000 GP phase 3) and the 1000 GP phase 3 genotypes was 99.59% (448,458/450,297). For the mtDNA markers, the concordance between the genotypes from the 859 markers (included and part of the 1000 GP phase 3) and the 1000 GP phase 3 genotypes was calculated as 99.84% (108,343/108,515). Overall, we confirmed that nearly all the genotypes captured by the DREAM array are accurate.

**Fig. 4.**
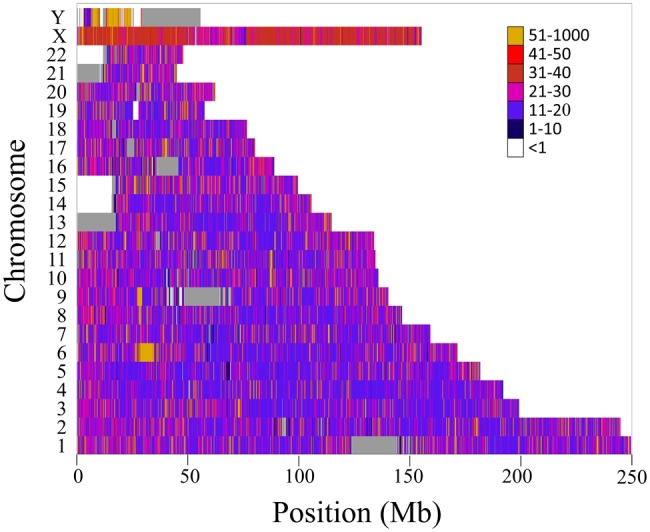
—SNP density in the DREAM microarray. The average numbers of DREAM SNPs per 100,000 nucleotides across the genome are color coded. Gaps in the assembly are shown in gray.

The SNP density across all chromosomes is shown in figure 4. 94% of the genome has a mean SNP density of 24.36, 33.34, 39.32 SNPs per 100 kilobases for the autosomes, X, and Y chromosomes, respectively. The remaining 6% correspond to the known gaps in the assembly of chromosomes 13, 14, 15, and 22. The short arm of chromosome 6 has the highest SNP density (56.41 SNPs per 100 kilobases) followed by the short arm of chromosome Y (50.53 SNPs per 100 kilobases).

DREAM’s potential to assist in ancient DNA studies was evaluated by calculating the number of ancient DNA genotypes for each ancient genome (supplementary fig. S3, Supplementary Material online). Of the 207 ancient human genomes used in the design, 201 genomes were well captured ($\overset{-}{L}$=22,641 SNPs) with 150 genomes having >100 SNPs. The captured genomes represented 12 out of 14 countries, excluding Montenegro (two genomes) and Lithuania (one genome), from time periods spanning 40,000 BC to 700 AD.

**Fig. 5.**
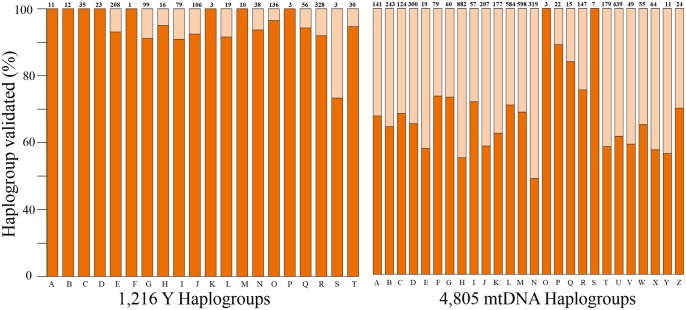
—Success rate in identifying Y-chromosomal (left) and mtDNA (right) haplogroups. The plots depict all known basal haplogroups (columns), the number of known subgroups in each haplogroup (top of each column), and the proportion of computationally validated subgroups.

DREAM’s ability to infer uniparental haplogroups was computationally assessed against the respective trees. DREAM markers identified 94% and 61% parental and maternal haplogroups, respectively (fig. 5). All the primary and secondary maternal haplogroups were detected.

### Assessing DREAM’s Abilities to Discern Population Structure

#### Comparing the Alternate Allele Frequency Distribution of Various Microarrays

Compared with whole genome data, allele frequencies (AF) in microarrays are typically shifted toward intermediate frequency levels (Elhaik et al. 2013), which led to the exploration of correction methods (Lachance and Tishkoff 2013). This is expected, provided that the majority of SNPs are private and that 1Mbp arrays that cover only 1% of the SNPs typically aim to capture common SNPs. None of the arrays we examined exhibited AF distribution similar to the 1000 GP, though they all roughly followed its trajectory (supplementary fig. S4, Supplementary Material online). Considering autosomal markers, DREAM had the highest proportion (70%) of common markers (AF > 0.05), after the Human Origins (77%), and its AF distribution resembled that of the HumanOmni2.5 array. Interestingly, despite its small size (1,443,399), the AF distribution of the Multi ethnic global array resembled that of the HumanOmni5 array for common markers. DREAM’s AF distribution in the X chromosome resembled the 1000 GP’s AF distribution after excluding rare variants (AF < 0.01), likely due to its enrichment with random markers. DREAM’s proportion of common markers (60%) was second only to the HumanOmni2.5 array (66%).

#### *Comparing the Genome-Wide F*_ST_ *Distribution of Various Microarrays*

The extent to which microarray technology is able to discern and identify subpopulations is of principal interest. *F*_ST_ is a measure of differentiation whereby the genetic variation of the subpopulation is measured relative to the total population (Wright 1951). Here, we employed data from the 1000 GP CEU, YRI, and CHB to calculate *F*_ST_ in DREAM and comparative arrays as in Elhaik (2012). DREAM produced the highest proportion of high-*F*_ST_ autosomal and X chromosomal alleles compared with other arrays (supplementary fig. S5, Supplementary Material online). The Multi ethnic global array had the second lowest *F*_ST_ values after the HumanOmni 5, which can be explained by the high proportion of rare SNPs they shared. The autosomes and X-chromosomal SNPs of the comparative arrays had significantly lower *F*_ST_ values (Kolmogorov–Smirnov goodness-of-fit test, *P *<* *0.001) than DREAM’s due to the high fraction of rare SNPs in these arrays. The magnitude of the differences between the *F*_ST_ values of these arrays was also large for autosomal (area overlap 69–77%, Cohen’s d 0.23–0.3) and X-chromosomal SNPs (area overlap 74–84%, Cohen’s d 0.17–0.26). These results suggest a reduced ability of the competing arrays to elucidate ancient demographic processes (Kimura and Ota 1973; Watterson and Guess 1977).

#### Comparing the Identical by Descent of Various Microarrays

IBD and haplotype-based methods are widely used in population genetic studies. Since, IBD coverage depends on the choice of population, proportion of rare alleles, and the number of SNPs, we compared the ratio of the total IBD coverage of three populations, which exhibit similar proportion of rare alleles, to the number of SNPs of each microarray. A high ratio indicates higher IBD coverage per SNP (supplementary fig. S6, Supplementary Material online). DREAM has the highest ratio for all populations compared with other arrays, excepting the Human Origins array (FIN). HumanOmni5 has the lowest ratio suggesting that the choice of SNPs is suboptimal. This is evidenced by the mean IBD coverage of FINs, which is 295.4 M using HumanOmni5, 321.7 M using HumanOmni2.5, and 214.8 M using DREAM. All arrays have similar standard deviations, but after normalizing for their size both DREAM and the Human Origins array exhibit the highest standard deviations for all populations.

#### Comparing the Linkage Disequilibrium Patterns of Various Microarrays

Optimizing microarray coverage can be done by including a core SNP panel with essential markers and selecting the remaining SNPs strategically to optimize imputation efforts. Such microarray design would consist of a fewer SNPs in high LD, whereas a wasteful or robust design (depending on one’s point of view) would consist of a large number of SNPs in high LD. A comparison of the LD patterns of SNPs from the four 1000 GP populations, which overlapped with each of the five microarrays showed, that the Human Origins microarray had the smallest fraction of high LD markers followed closely by DREAM (supplementary fig. S7, Supplementary Material online). This is expected as the Human Origins largely consists of sparse ancient DNA SNPs, whereas DREAM consists of a high fraction of genic markers. The LD cumulative probability distributions of the remaining microarrays generally clustered together with markers of the multi ethnic global microarray exhibiting the highest LD.

#### Detecting Interbreeding with Neanderthal and Denisovan

DREAM’s ability to infer IBD with archaic hominins was evaluated by comparing the total IBD between worldwide individuals, Neanderthal, and Denisovan calculated using the complete 36 million SNPs (1000 GP data set) and DREAM SNPs, representing 1.86% of the complete data set (supplementary table S6, Supplementary Material online). Total IBD region sizes were highly correlated (*N_Neanderthal_* = 450, *r_Neanderthal_* = 0.75, *N_Denisovan_* = 450, *r_Denisovan_* = 0.91) and exhibit similar between-population patterns in the two data sets.

#### Biogeographical Origins of Worldwide Populations

**Fig. 6.**
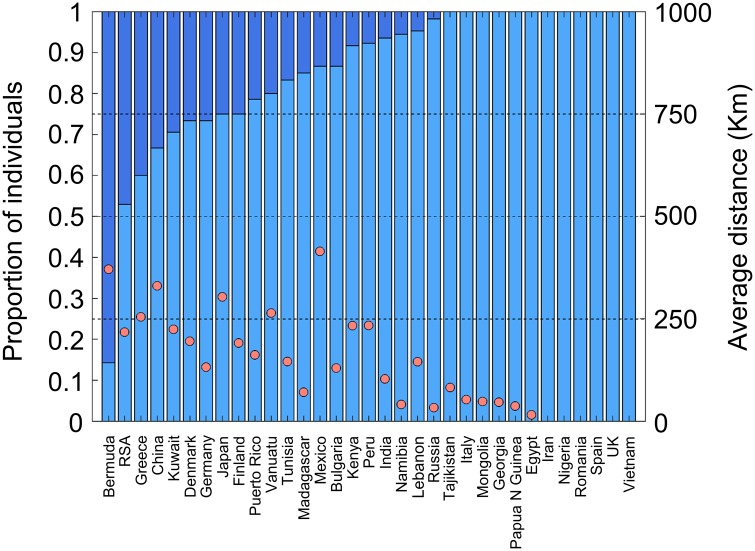
—GPS predictions of biogeographical affinities for worldwide 33 populations. The *x* axis illustrates populations represented by a vertical stacked column indicating the proportion of individuals predicted within 200 km of their country’s political borders (blue) and the remaining individuals (green). The average distance from the predicted location and true country of origin is indicated in red balls.

Prediction of biogeographical origins is obtained by converting genomic information into geographical coordinates. All biogeographical inferences were carried out using the geographic population structure (GPS) tool, which matches the admixture proportions of a test individual with those of *reference populations* known to have resided in a certain geographical region for a substantial period of time (Elhaik et al. 2014; Das et al. 2017). The efficacy of DREAM’s biogeographical predictions was assessed on 584 worldwide individuals from 33 countries (fig. 6 andsupplementary table S7, Supplementary Material online). DREAM placed the majority of individuals (88%) within <200 km from their country’s political borders, in line with Elhaik et al.’s (2014) report. For 36% of the countries, all the individuals were predicted within these extended boundaries. The average prediction distance from the true borders was 125 km, an improvement compared with previous studies (Das et al. 2016; Marshall et al. 2016). As expected, the accuracy decreased to 78% and 54% in the leave-one-out individual and -subpopulation analyses, respectively. There, in 15% and 6% 15% of the countries, respectively, all the individuals were predicted within the extended country’s boundaries in both cases and the average distances from the true borders were 230 and 473 km, respectively. These findings are similar or better than those reported by Elhaik et al. (2014) and reflect the choice of AIMs and the improvement made in the assembly of the *reference populations*.

Individual clustering by admixture proportions is an effective way to describe population structure (Marshall et al. 2016) and evaluate the ascertainment bias and the AIMs choice. An application of the graph-theoretical clustering technique NBR-Clust (Matta et al. 2016) to the admixture proportions of the Genographic individuals (supplementary fig. S1, Supplementary Material online) constructed a graph *G* (supplementary fig. S8, Supplementary Material online) with eight clusters, corresponding to the geographical regions that harbored the people. To examine whether individuals with higher *betweenness centrality* represent genetic mixtures with populations graphically adjacent to them, we created a *population graph G_P_* (supplementary fig. S9, Supplementary Material online) by merging individuals into their populations in graph *G*. Here too, nodes with notably high *betweenness* are the Bermudian, Tatarstan-Russian, Puerto Rican, Lima-Peruvian, North-Northeast Indian, and Antananarivo-Madagascan populations. These enlarged nodes lie on *cluster boundaries*. For example, the Madagascan node with high *betweenness* is adjacent to the Oceanic, East Asian, and his own African cluster, in support of recent reports of shared ancestry (Poetsch et al. 2013). North Indians are also adjacent to three clusters representing the Near East, East Asia, and their own Indian population, in agreement with recent studies that depicted these genomes as two-way mixture between West Eurasians and indigenous Andaman Islanders (Moorjani et al. 2013). Our findings are therefore consistent with the known history and demographics of the admixed populations and support the utility of the NBR-Clust framework to represent population structure. Further insights can be made by applying adjacency and graph distance information. We note that graph theoretic representation retains high-dimensional information that may be lost in performing 2D or 3D PCA for visualization.

#### Analysis of CNVs in Worldwide Populations

Sudmant et al. (2015) reported that the CNV distribution in human population can be used to reconstruct population structure. For example, the authors found that for deletions, the first two principal components distinguished Africans, West Eurasians, East Asians, and Oceanian populations with many other populations clustering with their continental populations. Similar trends were found for duplications, albeit with far less clarity. They also reported that African populations are broadly distinguished from non-African for either deletions or duplications. Our results reflect Sudmant et al.’s findings in that deletions largely allowed distinguishing regional populations, deletions identified a more coherent population structure than duplications (supplementary fig. S10, Supplementary Material online), and finally that Africans were largely separated from non-Africans for both CNV types.

## Conclusions

We designed, developed, validated, and assessed the DREAM microarray, an all-inclusive SNP genotyping chip dedicated to genetic anthropology and genealogy, forensics, and personalized medicine. DREAM can be used to study the genetic relationships between ancient humans, archaic hominins, and modern humans as well as to improve our understanding of human migratory history, adaptations, and the molecular mechanisms that regulate forensic-relevant traits. By comparing the MAF and *F*_ST_ distributions of the DREAM array to those of the 1000 GP and commercially available arrays, we demonstrated DREAM’s ability to differentiate populations within global data sets. Lastly, we demonstrated the biogeographical accuracy of DREAM and its potential ability to infer CNVs. We expect that the use of the DREAM in genealogy and research will expand our knowledge of our species. 

## Supplementary Material

Supplementary data are available at *Genome Biology and Evolution* online.

## Author Contributions

E.E. initiated the study and designed DREAM with Y.L. and T.W. M.L.B. supervised the work. E.E., L.Y., A.A., M.H., D.A., G.V., G.E., and U.E. carried out the analysis. E.E. and L.Y. wrote the manuscript. All authors approved the manuscript.

## Supplementary Material

Supplementary Figures and Tables

## References

[evx237-B1] Albrechtsen A, 2010 Ascertainment biases in SNP chips affect measures of population divergence. Mol Biol Evol. 27(11):2534–2547.http://dx.doi.org/10.1093/molbev/msq148 20558595 10.1093/molbev/msq148 PMC3107607

[evx237-B2] Allentoft ME, 2015 Population genomics of Bronze Age Eurasia. Nature 522(7555):167–172.http://dx.doi.org/10.1038/nature14507 26062507 10.1038/nature14507

[evx237-B4] Bastian M, 2009 Gephi: an open source software for exploring and manipulating networks. International AAAI Conference on Weblogs and Social Media. p. 361–362. [cited 2017 Jul 16] Available from: http://www.aaai.org/ocs/index.php/ICWSM/09/paper/view/154/.

[evx237-B5] Benjamini Y, Hochberg Y. 1995 Controlling the false discovery rate: a practical and powerful approach to multiple testing. J R Stat Soc B 57:289–300.

[evx237-B6] Browning BL, Browning SR. 2013 Improving the accuracy and efficiency of identity by descent detection in population data. Genetics 194(2):459–471.http://dx.doi.org/10.1534/genetics.113.150029 23535385 10.1534/genetics.113.150029 PMC3664855

[evx237-B7] Crawford DC, 2014 eMERGEing progress in genomics—the first seven years. Front Genet. 5:184. 24987407 10.3389/fgene.2014.00184 PMC4060012

[evx237-B8] Danecek P, 2011 The variant call format and VCFtools. Bioinformatics 27(15):2156–2158.http://dx.doi.org/10.1093/bioinformatics/btr330 21653522 10.1093/bioinformatics/btr330 PMC3137218

[evx237-B9] Daneshjou R, 2014 Genetic variant in folate homeostasis associated with lower warfarin dose in African Americans. Blood 124(14):2298–2305.http://dx.doi.org/10.1182/blood-2014-04-568436 25079360 10.1182/blood-2014-04-568436 PMC4183989

[evx237-B10] Das R, 2016 Localizing Ashkenazic Jews to primeval villages in the ancient Iranian lands of Ashkenaz. Genome Biol Evol. 8(4):1132–1149.http://dx.doi.org/10.1093/gbe/evw046 26941229 10.1093/gbe/evw046 PMC4860683

[evx237-B11] Das R, 2017 The origins of Ashkenaz, Ashkenazic Jews, and Yiddish. Front Genet. 8. 10.3389/fgene.2017.00087 PMC5478715 28680441

[evx237-B12] DePristo MA, 2011 A framework for variation discovery and genotyping using next-generation DNA sequencing data. Nat Genet. 43(5):491–498.http://dx.doi.org/10.1038/ng.806 21478889 10.1038/ng.806 PMC3083463

[evx237-B13] Durbin RM, 2010 A map of human genome variation from population-scale sequencing. Nature 467(7319):1061–1073.http://dx.doi.org/10.1038/nature09534 20981092 10.1038/nature09534 PMC3042601

[evx237-B14] Elhaik E. 2012 Empirical distributions of *F*_ST_ from large-scale human polymorphism data. PLoS One 7(11):e49837. 23185452 10.1371/journal.pone.0049837 PMC3504095

[evx237-B15] Elhaik E, 2013 The GenoChip: a new tool for genetic anthropology. Genome Biol Evol. 5(5):1021–1031.http://dx.doi.org/10.1093/gbe/evt066 23666864 10.1093/gbe/evt066 PMC3673633

[evx237-B16] Elhaik E, 2014 Geographic population structure analysis of worldwide human populations infers their biogeographical origins. Nat Commun. 5. 10.1038/ncomms4513 PMC4007635 24781250

[evx237-B17] Elhaik E, Graur D. 2013 IsoPlotter+: a tool for studying the compositional architecture of genomes. ISRN Bioinformatics 2013:6. 10.1155/2013/725434 PMC4393066 25937951

[evx237-B18] Enoch MA, 2006 Using ancestry-informative markers to define populations and detect population stratification. J Psychopharmacol. 20(4_suppl):19–26. 10.1177/1359786806066041 16785266

[evx237-B19] Fan S, 2016 Going global by adapting local: a review of recent human adaptation. Science 354(6308):54–59.http://dx.doi.org/10.1126/science.aaf5098 27846491 10.1126/science.aaf5098 PMC5154245

[evx237-B20] Fardon R. 2012 The Sage handbook of social anthropology. Thousand Oaks (CA): SAGE Publications.

[evx237-B21] Freeman JL, 2006 Copy number variation: new insights in genome diversity. Genome Res. 16(8):949–961.http://dx.doi.org/10.1101/gr.3677206 16809666 10.1101/gr.3677206

[evx237-B22] Fu Q, 2014 Genome sequence of a 45, 000-year-old modern human from western Siberia. Nature 514(7523):445–449.http://dx.doi.org/10.1038/nature13810 25341783 10.1038/nature13810 PMC4753769

[evx237-B23] Gamba C, 2014 Genome flux and stasis in a five millennium transect of European prehistory. Nat Commun. 5:5257. 25334030 10.1038/ncomms6257 PMC4218962

[evx237-B24] Green RE, 2010 A draft sequence of the Neandertal genome. Science 328(5979):710–722.http://dx.doi.org/10.1126/science.1188021 20448178 10.1126/science.1188021 PMC5100745

[evx237-B25] Haak W, 2015 Massive migration from the steppe was a source for Indo-European languages in Europe. Nature 522(7555):207–211.http://dx.doi.org/10.1038/nature14317 25731166 10.1038/nature14317 PMC5048219

[evx237-B26] Hedges LV. 1981 Distribution theory for Glass’s estimator of effect size and related estimators. J Educ Behav Stat. 6(2):107–128.

[evx237-B27] illumina. 2010 The power of intelligent SNP selection [cited 2017 Jul 16]. Available from: https://www.illumina.com/content/dam/illumina-marketing/documents/products/technotes/technote_intelligent_snp_selection.pdf.

[evx237-B28] illumina. 2013 HumanOmni2.5-8 v1.0 minor allele frequencies [cited 2017 Jun 16]. Available from: ftp://webdata2: webdata2@ussd-ftp.illumina.com/MyIllumina/af2dd135-8455-41f2-95ea-29e09b067283/HumanOmni2.5-8v1_C_MAF.zip.

[evx237-B29] illumina. 2015 HumanOmni5-Quad v1.0 minor allele frequencies [cited 2017 Jun 16]. Available from: https://support.illumina.com/array/array_kits/humanomni5-4-beadchip-kit/downloads.html.

[evx237-B30] illumina. 2016 Infinium multi-ethnic Global-8 v1.0 population reports (MAF, copy numbers) [cited 2017 Jun 16]. Available from: https://www.illumina.com/products/by-type/microarray-kits/infinium-multi-ethnic-global.html.

[evx237-B501] Jensen LJ, 2009 STRING 8—a global view on proteins and their functional interactions in 630 organisms. Nucleic Acids Res. 37:D412–416. 10.1093/nar/gkn760 PMC2686466 18940858

[evx237-B31] Jobling M, 2013 Human evolutionary genetics: origins, peoples & disease. New York: Garland Science.

[evx237-B32] Johnson JA. 2008 Ethnic differences in cardiovascular drug response: potential contribution of pharmacogenetics. Circulation 118(13):1383–1393.http://dx.doi.org/10.1161/CIRCULATIONAHA.107.704023 18809808 10.1161/CIRCULATIONAHA.107.704023 PMC2730023

[evx237-B33] Kayser M. 2015 Forensic DNA phenotyping: predicting human appearance from crime scene material for investigative purposes. Forensic Sci Int Genet. 18:33–48.http://dx.doi.org/10.1016/j.fsigen.2015.02.003 25716572 10.1016/j.fsigen.2015.02.003

[evx237-B34] Keller A, 2012 New insights into the Tyrolean Iceman’s origin and phenotype as inferred by whole-genome sequencing. Nat Commun. 3:698. 22426219 10.1038/ncomms1701

[evx237-B35] Kimura M, Ota T. 1973 The age of a neutral mutant persisting in a finite population. Genetics 75(1):199–212. 4762875 10.1093/genetics/75.1.199 PMC1212997

[evx237-B36] Lachance J, Tishkoff SA. 2013 SNP ascertainment bias in population genetic analyses: why it is important, and how to correct it. Bioessays 35(9):780–786.http://dx.doi.org/10.1002/bies.201300014 23836388 10.1002/bies.201300014 PMC3849385

[evx237-B37] Landrum MJ, 2016 ClinVar: public archive of interpretations of clinically relevant variants. Nucleic Acids Res. 44(D1):D862–D868. 26582918 10.1093/nar/gkv1222 PMC4702865

[evx237-B38] Lazaridis I, 2014 Ancient human genomes suggest three ancestral populations for present-day Europeans. Nature 513(7518):409–413.http://dx.doi.org/10.1038/nature13673 25230663 10.1038/nature13673 PMC4170574

[evx237-B39] Li H. 2011 A statistical framework for SNP calling, mutation discovery, association mapping and population genetical parameter estimation from sequencing data. Bioinformatics 27(21):2987–2993.http://dx.doi.org/10.1093/bioinformatics/btr509 21903627 10.1093/bioinformatics/btr509 PMC3198575

[evx237-B40] Li H, Durbin R. 2009 Fast and accurate short read alignment with Burrows-Wheeler transform. Bioinformatics 25(14):1754–1760.http://dx.doi.org/10.1093/bioinformatics/btp324 19451168 10.1093/bioinformatics/btp324 PMC2705234

[evx237-B41] Li H, 2009 The sequence Alignment/Map format and SAMtools. Bioinformatics 25(16):2078–2079.http://dx.doi.org/10.1093/bioinformatics/btp352 19505943 10.1093/bioinformatics/btp352 PMC2723002

[evx237-B42] Llorente MG, 2015 Ancient Ethiopian genome reveals extensive Eurasian admixture throughout the African continent. Science 350(6262):820–822.http://dx.doi.org/10.1126/science.aad2879 26449472 10.1126/science.aad2879

[evx237-B43] Lu Y, 2011 Technical design document for a SNP array that is optimized for population genetics [cited 2017 Jun 16]. Available from: ftp://ftp.cephb.fr/hgdp_supp10/8_12_2011_Technical_Array_Design_Document.pdf.

[evx237-B44] Marshall S, 2016 Reconstructing Druze population history. Sci Rep. 6:35837. 27848937 10.1038/srep35837 PMC5111078

[evx237-B45] Matta J, 2016. Robust graph-theoretic clustering approaches using node-based resilience measures. Data Mining (ICDM), 2016 IEEE 16th International Conference on IEEE. p. 320–329.

[evx237-B46] McKenna A, 2010 The genome analysis toolkit: a MapReduce framework for analyzing next-generation DNA sequencing data. Genome Res. 20(9):1297–1303.http://dx.doi.org/10.1101/gr.107524.110 20644199 10.1101/gr.107524.110 PMC2928508

[evx237-B47] McLaren W, 2016 The Ensembl variant effect predictor. Genome Biol. 17(1):122 http://dx.doi.org/10.1186/s13059-016-0974-4 27268795 10.1186/s13059-016-0974-4 PMC4893825

[evx237-B48] Meyer M, 2012 A high-coverage genome sequence from an archaic Denisovan individual. Science 338(6104):222–226.http://dx.doi.org/10.1126/science.1224344 22936568 10.1126/science.1224344 PMC3617501

[evx237-B49] Moorjani P, 2013 Genetic evidence for recent population mixture in India. Am J Hum Genet. 93(3):422–438.http://dx.doi.org/10.1016/j.ajhg.2013.07.006 23932107 10.1016/j.ajhg.2013.07.006 PMC3769933

[evx237-B50] Olalde I, 2014 Derived immune and ancestral pigmentation alleles in a 7, 000-year-old Mesolithic European. Nature 507(7491):225–228.http://dx.doi.org/10.1038/nature12960 24463515 10.1038/nature12960 PMC4269527

[evx237-B51] Poetsch M, 2013 Determination of population origin: a comparison of autosomal SNPs, Y-chromosomal and mtDNA haplogroups using a Malagasy population as example. Eur J Hum Genet. 21(12):1423–1428.http://dx.doi.org/10.1038/ejhg.2013.51 23612573 10.1038/ejhg.2013.51 PMC3831070

[evx237-B52] Popejoy AB, Fullerton SM. 2016 Genomics is failing on diversity. Nature 538(7624):161. http://dx.doi.org/10.1038/538161a 27734877 10.1038/538161a PMC5089703

[evx237-B53] Purcell S, 2007 PLINK: a tool set for whole-genome association and population-based linkage analyses. Am J Hum Genet. 81(3):559–575.http://dx.doi.org/10.1086/519795 17701901 10.1086/519795 PMC1950838

[evx237-B54] Racimo F, 2017 Signatures of archaic adaptive introgression in present-day human populations. Mol Biol Evol. 34(2):296–317. 27756828 10.1093/molbev/msw216 PMC5400396

[evx237-B55] Raghavan M, 2014 Upper Palaeolithic Siberian genome reveals dual ancestry of Native Americans. Nature 505(7481):87–91.http://dx.doi.org/10.1038/nature12736 24256729 10.1038/nature12736 PMC4105016

[evx237-B56] Sawyer S, 2015 Nuclear and mitochondrial DNA sequences from two Denisovan individuals. Proc Natl Acad Sci U S A. 112(51):15696–15700. 26630009 10.1073/pnas.1519905112 PMC4697428

[evx237-B57] Schiffels S, 2016 Iron Age and Anglo-Saxon genomes from East England reveal British migration history. Nat Commun. 7:10408. 26783965 10.1038/ncomms10408 PMC4735688

[evx237-B58] Scott SA, 2008 Warfarin pharmacogenetics: *CYP2C9* and *VKORC1* genotypes predict different sensitivity and resistance frequencies in the Ashkenazi and Sephardi Jewish populations. Am J Hum Genet. 82(2):495–500.http://dx.doi.org/10.1016/j.ajhg.2007.10.002 18252229 10.1016/j.ajhg.2007.10.002 PMC2427171

[evx237-B59] Seguin-Orlando A, 2014 Genomic structure in Europeans dating back at least 36, 200 years. Science 346(6213):1113–1118.http://dx.doi.org/10.1126/science.aaa0114 25378462 10.1126/science.aaa0114

[evx237-B60] Shadyab AH, LaCroix AZ. 2015 Genetic factors associated with longevity: a review of recent findings. Ageing Res Rev. 19:1–7.http://dx.doi.org/10.1016/j.arr.2014.10.005 25446805 10.1016/j.arr.2014.10.005

[evx237-B61] Sissung TM, 2010 Clinical pharmacology and pharmacogenetics in a genomics era: the DMET platform. Pharmacogenomics 11(1):89–103.http://dx.doi.org/10.2217/pgs.09.154 20017675 10.2217/pgs.09.154 PMC6448402

[evx237-B62] Skoglund P, 2014 Genomic diversity and admixture differs for Stone-Age Scandinavian foragers and farmers. Science 344(6185):747–750.http://dx.doi.org/10.1126/science.1253448 24762536 10.1126/science.1253448

[evx237-B63] Sudmant PH, 2013 Evolution and diversity of copy number variation in the great ape lineage. Genome Res. 23(9):1373–1382.http://dx.doi.org/10.1101/gr.158543.113 23825009 10.1101/gr.158543.113 PMC3759715

[evx237-B64] Sudmant PH, 2015 Global diversity, population stratification, and selection of human copy-number variation. Science 349(6253):aab3761. 26249230 10.1126/science.aab3761 PMC4568308

[evx237-B65] Thermo Fisher Scientific. 2015 Axiom™ CNV summary tools software [cited 2017 Jun 17]. Available from: https://www.thermofisher.com/uk/en/home/life-science/microarray-analysis/microarray-analysis-instruments-software-services/microarray-analysis-software/axiom-cnv-summary-tools-software.html#1_1.

[evx237-B66] Thermo Fisher Scientific. 2017 Axiom™ genotyping solution. Data analysis guide [cited 2017 Jun 17]. Available from: http://tools.thermofisher.com/content/sfs/manuals/axiom_genotyping_solution_analysis_guide.pdf.

[evx237-B67] Thermo Fisher Scientific. Axiom^®^ biobank genotyping solution [cited 2017 Jun 16]. Available from: https://tools.thermofisher.com/content/sfs/brochures/axiom_biobank_solution_brochure.pdf.

[evx237-B68] Watterson GA, Guess HA. 1977 Is the most frequent allele the oldest? Theor Popul Biol. 11(2):141–160. 867285 10.1016/0040-5809(77)90023-5

[evx237-B69] Webster T, 2013 Detection of CNV gains and losses with Affymetrix^®^ Axiom^®^ arrays [cited 2017 Jun 17]. Available from: http://www.ashg.org/2013meeting/abstracts/fulltext/f130121341.htm.

[evx237-B70] Whirl-Carrillo M, 2012 Pharmacogenomics knowledge for personalized medicine. Clin Pharmacol Ther. 92(4):414–417.http://dx.doi.org/10.1038/clpt.2012.96 22992668 10.1038/clpt.2012.96 PMC3660037

[evx237-B71] Wright S. 1951 The genetical structure of populations. Ann Eugenics 15(4):323–354. 10.1111/j.1469-1809.1949.tb02451.x 24540312

[evx237-B72] Yusuf S, Wittes J. 2016 Interpreting geographic variations in results of randomized, controlled trials. N Engl J Med. 375(23):2263–2271. 27959693 10.1056/NEJMra1510065

[evx237-B73] Zarrei M, 2015 A copy number variation map of the human genome. Nat Rev Genet. 16(3):172–183.http://dx.doi.org/10.1038/nrg3871 25645873 10.1038/nrg3871

