# Supplementary material for: The Diversity of REcent and Ancient huMan (DREAM): A New Microarray for Genetic Anthropology and Genealogy, Forensics, and Personalized Medicine
Source: Genome Biol Evol. 2017 Nov 20;9(12):3225–37. doi: 10.1093/gbe/evx237 (PMC5726468; doi:10.1093/gbe/evx237)
Supplement: Supplementary Figures and Tables [file evx237_supp.zip › DREAM - Supp.docx]

The **D**iversity of **RE**cent and **A**ncient hu**M**an (DREAM): a new microarray for genetic anthropology and genealogy, forensics, and personalized medicine

**Supplementary information**

Text S1: Y-chromosomal and mtDNA SNP inclusion

## Y chromosomal markers

Our goal was to infer all 1,216 Y haplogroups. For that, we obtained the 17,056 SNPs of the most updated Y tree (International Society of Genetic Genealogy 2015) and those included in the GenoChip (13,611), reported to identify 82% of all paternal haplogroups (Elhaik et al. 2013). Designing probesets for Y chromosome markers is challenging due to the high homology between non-PAR (Pseudoautosomal Regions) Y sequences and other chromosomes, especially the X chromosome. High homology results in cross hybridization between unintended chromosomes and the non-PAR Y probe sequences, and interferes with the genotyping of the intended Y SNP. As before, we addressed this problem in two ways: first by excluding probesets based sequence properties, and second by evaluating the probeset clusters after genotyping. Probesets were excluded if their 30mer sequence was not unique, or if there was potential for interfering adjacent markers within the 30mer sequence. A marker was considered to interfere if it was within 20 bases from the ligation site where the target marker is differentiated and has MAF ≥ 0.5% according to 1000 GP phase 1. Exceptions were made for Y markers that appear to be working based on performance on previous arrays. Initially, 28,199 probesets interrogating 18,091 markers were curated for further analyses.

Here too, probesets were sorted based on the genotype clusters produced by the 139 1000 GP individuals (see *SNP validation*). If the non-PAR Y region is reasonably distinct, the center of the genotype clusters of male samples will be distinguished from the female samples. Probesets that did reasonably resolve genotype clusters produced by the male and female samples were excluded. 631 probesets that called the alternative allele in over 98% of the cases likely due to cross hybridization with high homology region were also removed. Overall, 20,458 probesets interrogating 13,576 Y markers were considered reliable and included in DREAM.

## MtDNA markers

Our goal was to infer at least all the primary and secondary basal mtDNA haplogroups out of the 4,805 halogroups (mtDNA community 2016). We obtained all known mtDNA markers and prioritized those that allow inferring the basal haplogroups. Designing probesets to interrogate mitochondrial markers is challenging due to the immense variability and mutation density of its genome. We addressed this problem in two ways: first by upfront exclusion of the probesets from the array based sequence properties, and second by visual evaluation of probeset cluster properties after genotyping Probesets were excluded from the array design if the 30mer sequence was not unique or there was potential for interfering adjacent markers within the 30mer sequence. A marker was considered to interfere if it was within 20 bases from the ligation site where the target marker is differentiated and has a minor allele frequency (MAF) >=1% according to the 1000 GP phase 1. Exceptions were made for markers that appear to be working based on performance on previous arrays. Initially, 1,593 probesets interrogating 1304 markers were curated for further analyses.

Visual evaluation of these 1,593 probesets was based on genotyping 139 1000 GP individuals (see *SNP validation*) and probesets producing genotype cluster patterns consistent with interfering neighboring mutations were excluded. Twelve more probesets that called the alternative allele in over 98% of the cases likely due to cross hybridization with high homology region were removed. Overall, 1,385 probesets interrogating 1,172 markers were included in DREAM.

Supplementary figure legend

## Figure S1

Admixture analysis of worldwide populations and subpopulations. Admixture analysis was performed for *K*=9. For brevity, subpopulations were collapsed. The x axis represents individuals from populations sorted according to their reported ancestries. Each individual is represented by a vertical stacked column of color-coded admixture proportions that reflects genetic contributions from putative ancestral populations. Although the analyses was done using 18% of the SNPs as in (Elhaik et al. 2014, Figure 1) the results are remarkably similar.


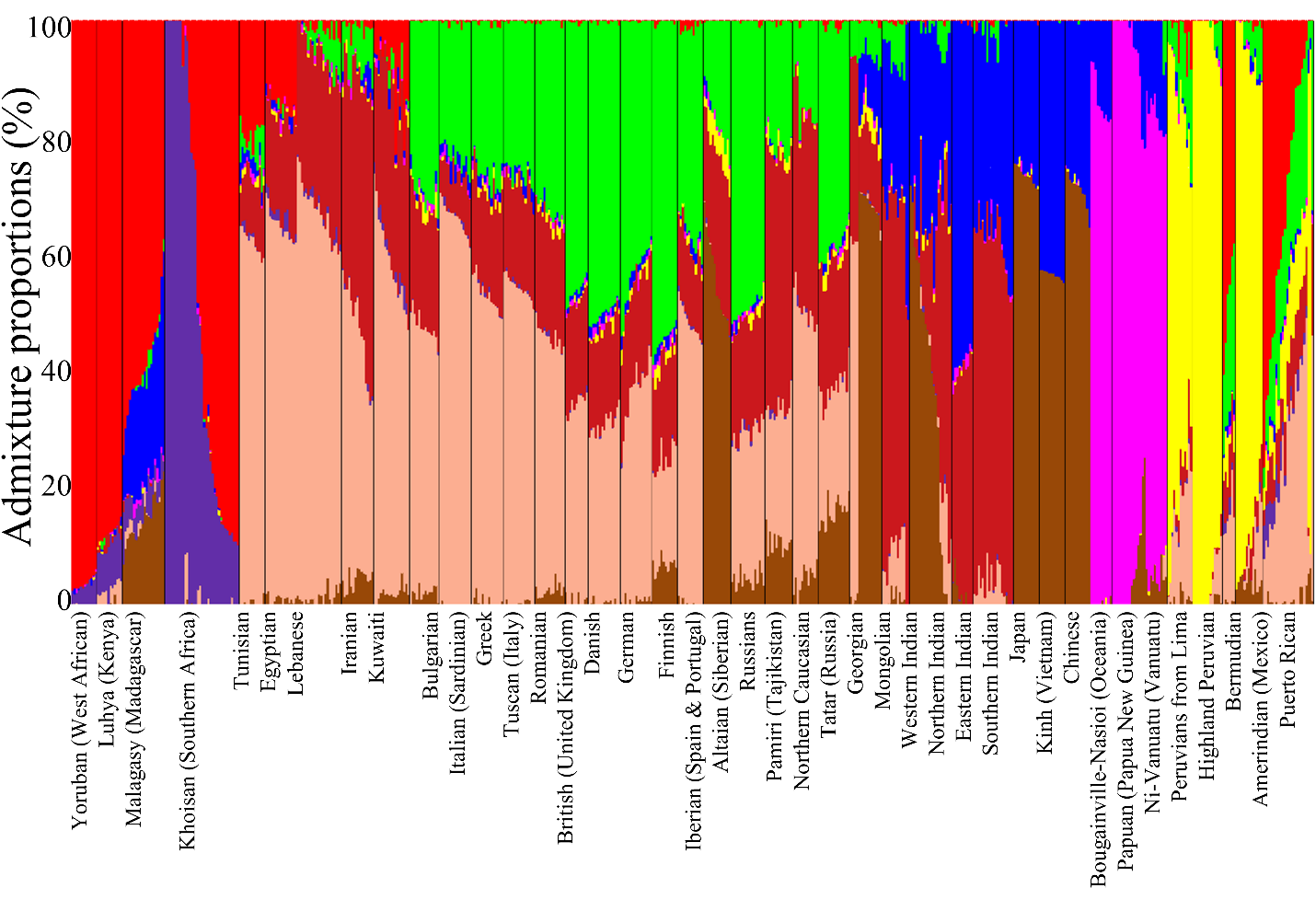


## Figure S2

Chromosomal view of DREAM’s CNV distribution. The distribution of 351 CNVs is shown per chromosome. CNVs range is shown in red with triangle symbol indicating the starting position. We note that due to their small sizes the range of some CNVs cannot be seen.


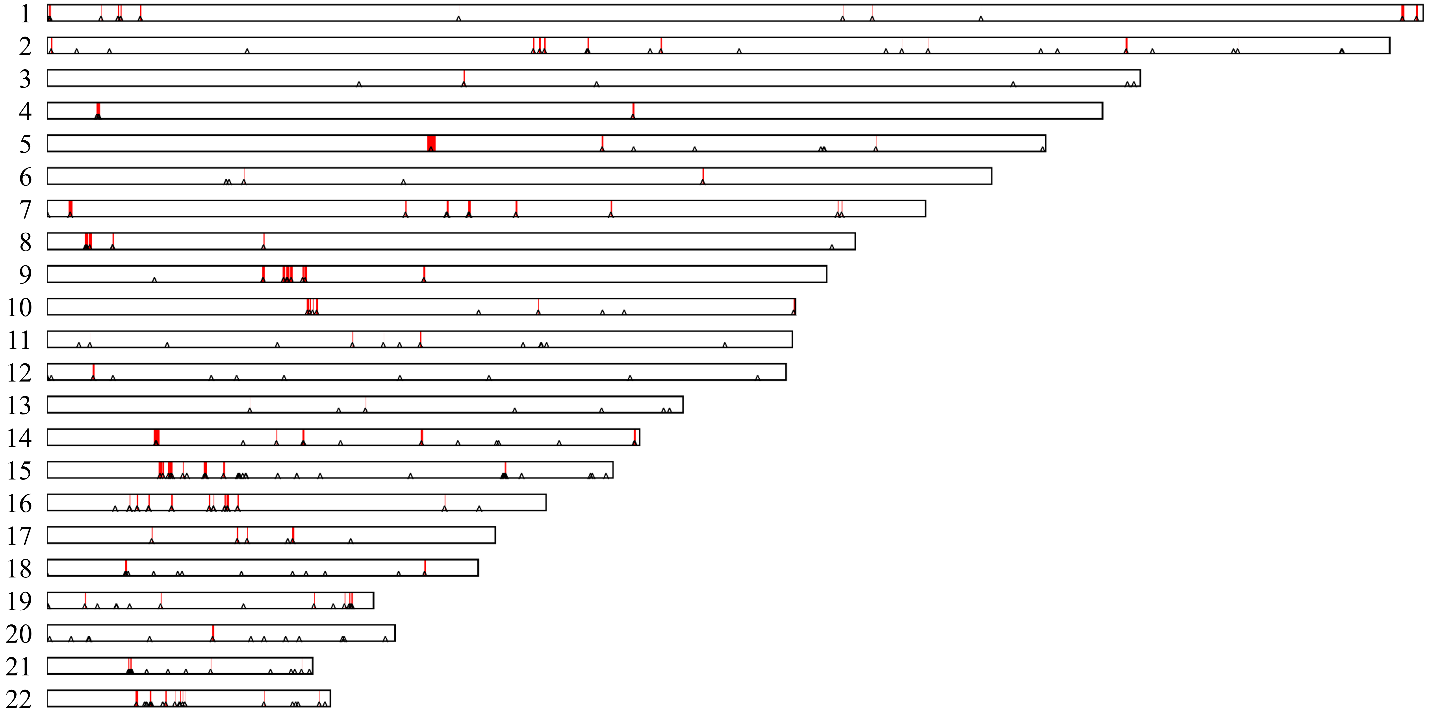


## Figure S3

Markers shared between aDNA genomes and the DREAM array. Points indicate the number of markers shared between an aDNA genome and the DREAM array. Ancient genomes are clustered by country with the exception of the last two entries. There, only the eight low-coverage Neanderthal and Denisovan genomes are shown.


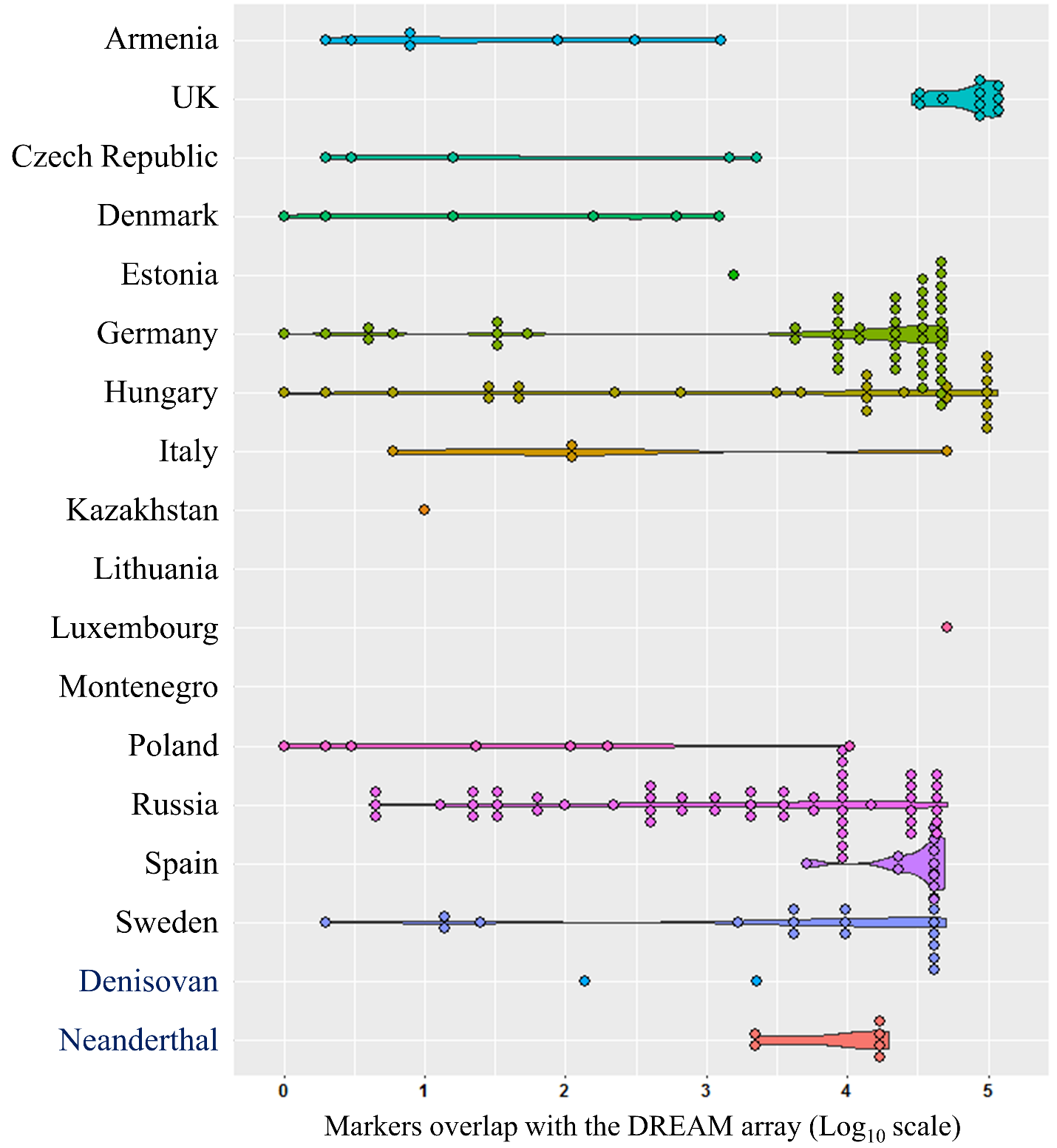


## Figure S4

Alternative allele frequency distributions for autosomal and X-chromosomal (inset) SNPs based on 1000 GP data. AF distributions are compared across seven subsets: all 1000 GP and rare 1000 GP SNPs (1000 GP^–^), SNPs included in five arrays: HumanOmni 5 (Illumina), Multi ethnic global (Illumina), the HumanOmni 2.5 (Illumina), Human Origins (Thermo Fisher), and DREAM (Thermo Fisher).


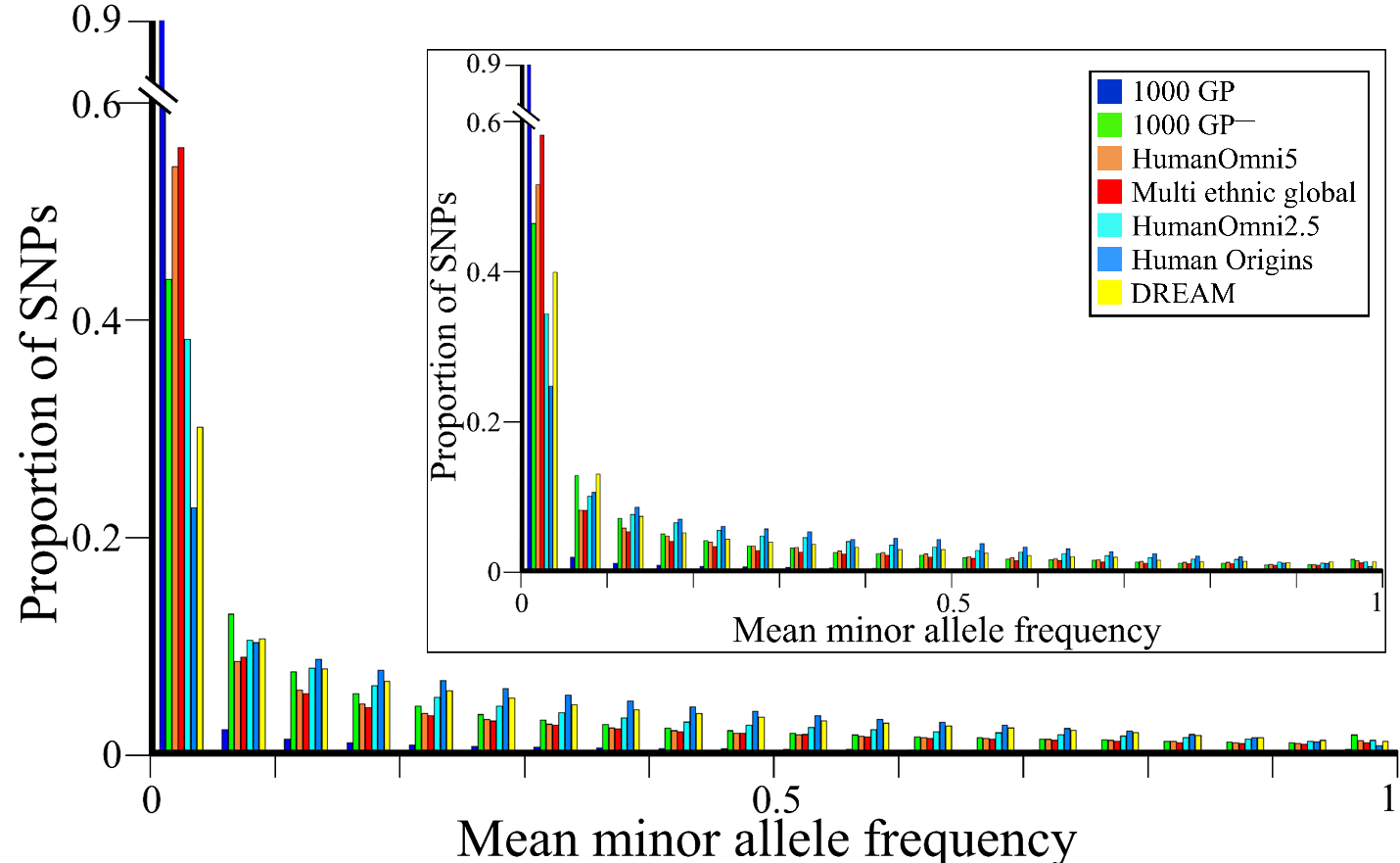


## Figure S5

Distribution of locus-specific *F_ST_* in three continental populations (CEU, YTI, CHB) based on 1000 GP data. *F_ST_* values were obtained for autosomal and X-chromosomal (inset) SNPs. *F_ST_* distributions are compared across five microarrays: HumanOmni 5 (Illumina), Multi ethnic global (Illumina), the HumanOmni 2.5 (Illumina), Human Origins (Thermo Fisher), and DREAM (Thermo Fisher). The histograms show bin distribution as indicated on the x axis and the cumulative distribution (line).


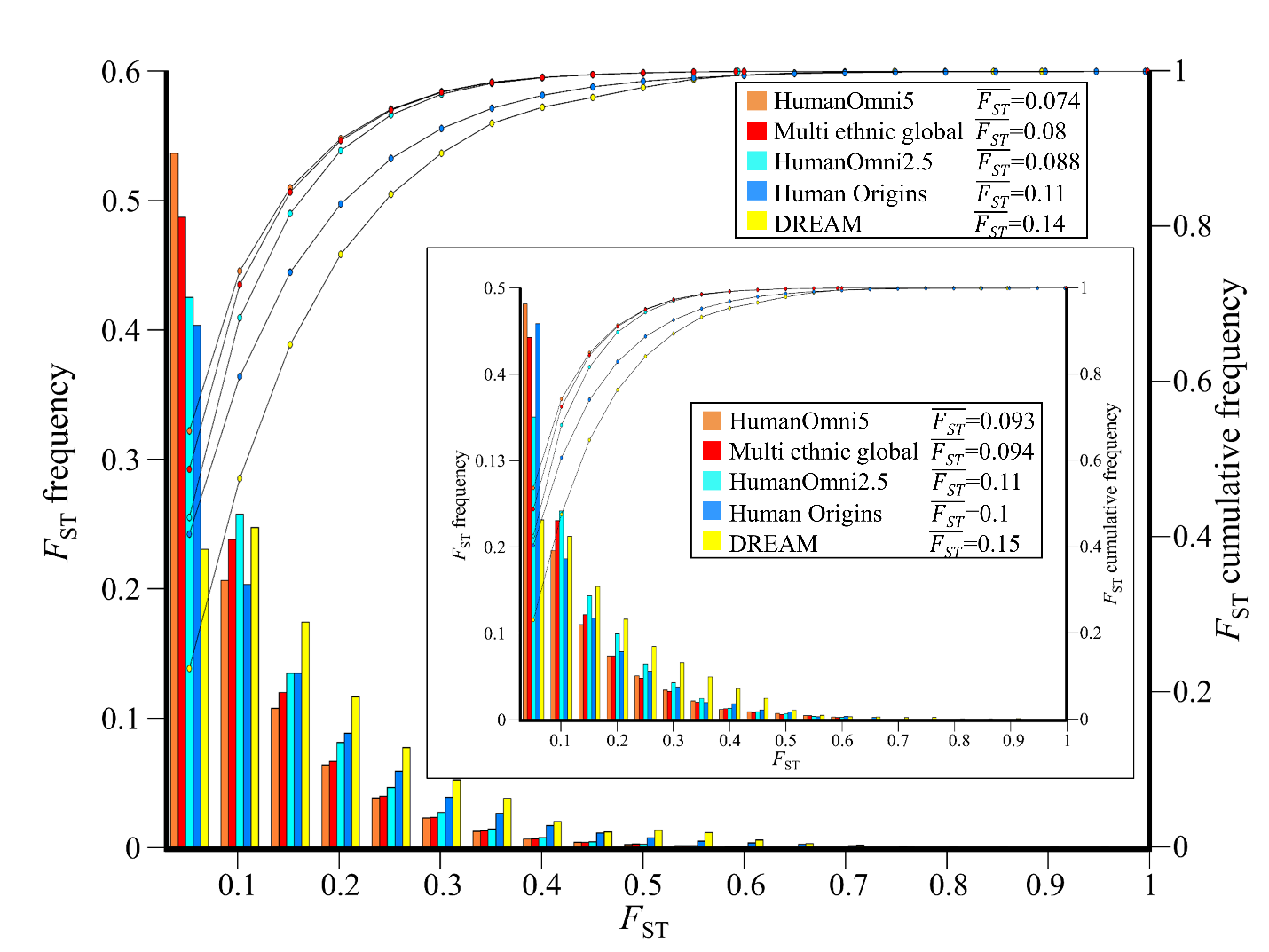


## Figure S6

A comparison of the proportion of IBD coverage to number of SNPs across populations and microarrays. The bar plot shows the mean and standard deviation of the Log_10_ ratio of IBD coverage to microarray size for autosomal SNPs.


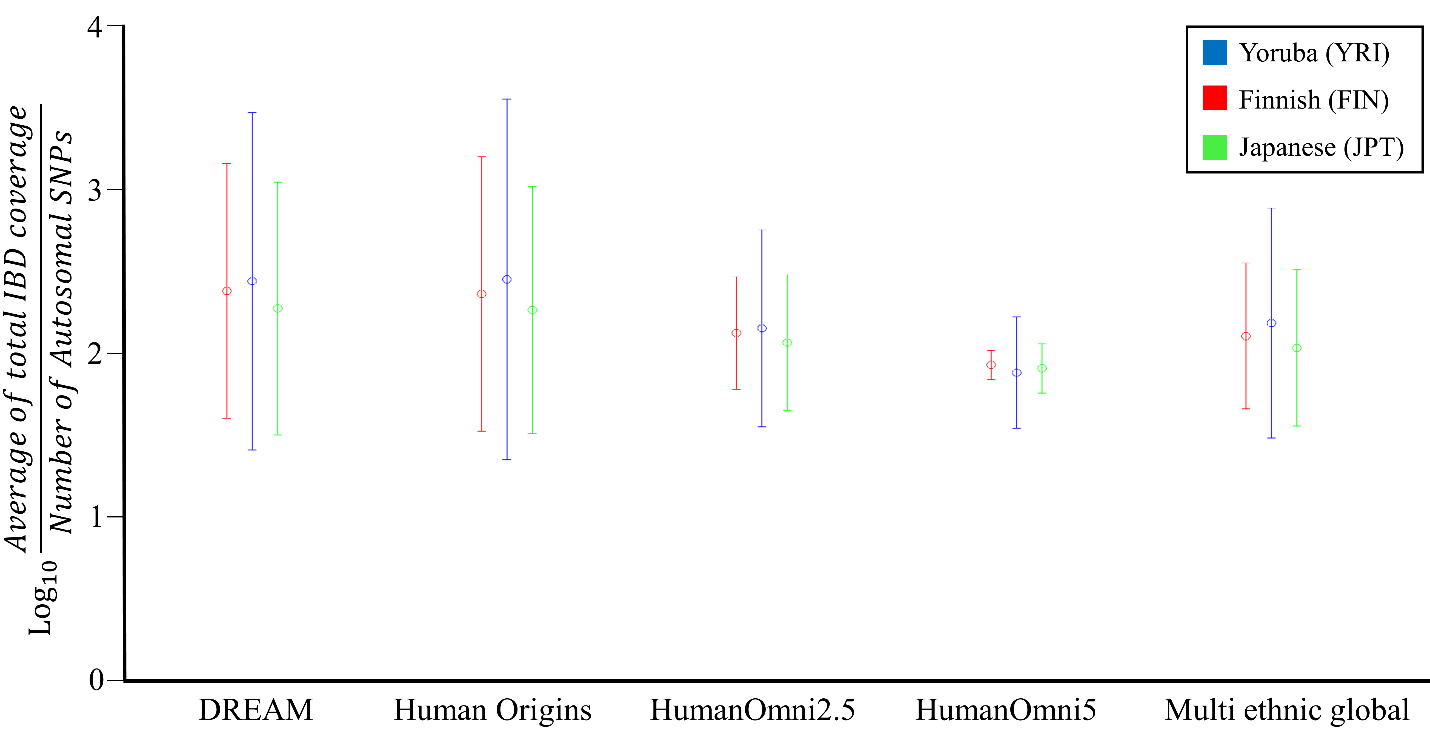


## Figure S7

**LD patterns for 1000 GP populations calculated for the microarray SNPs**. The cumulative probability distribution of LD (*r^2^*) is shown for: Finnish (A), Yoruba (B), Japanese (C), and Puerto Ricans (D).


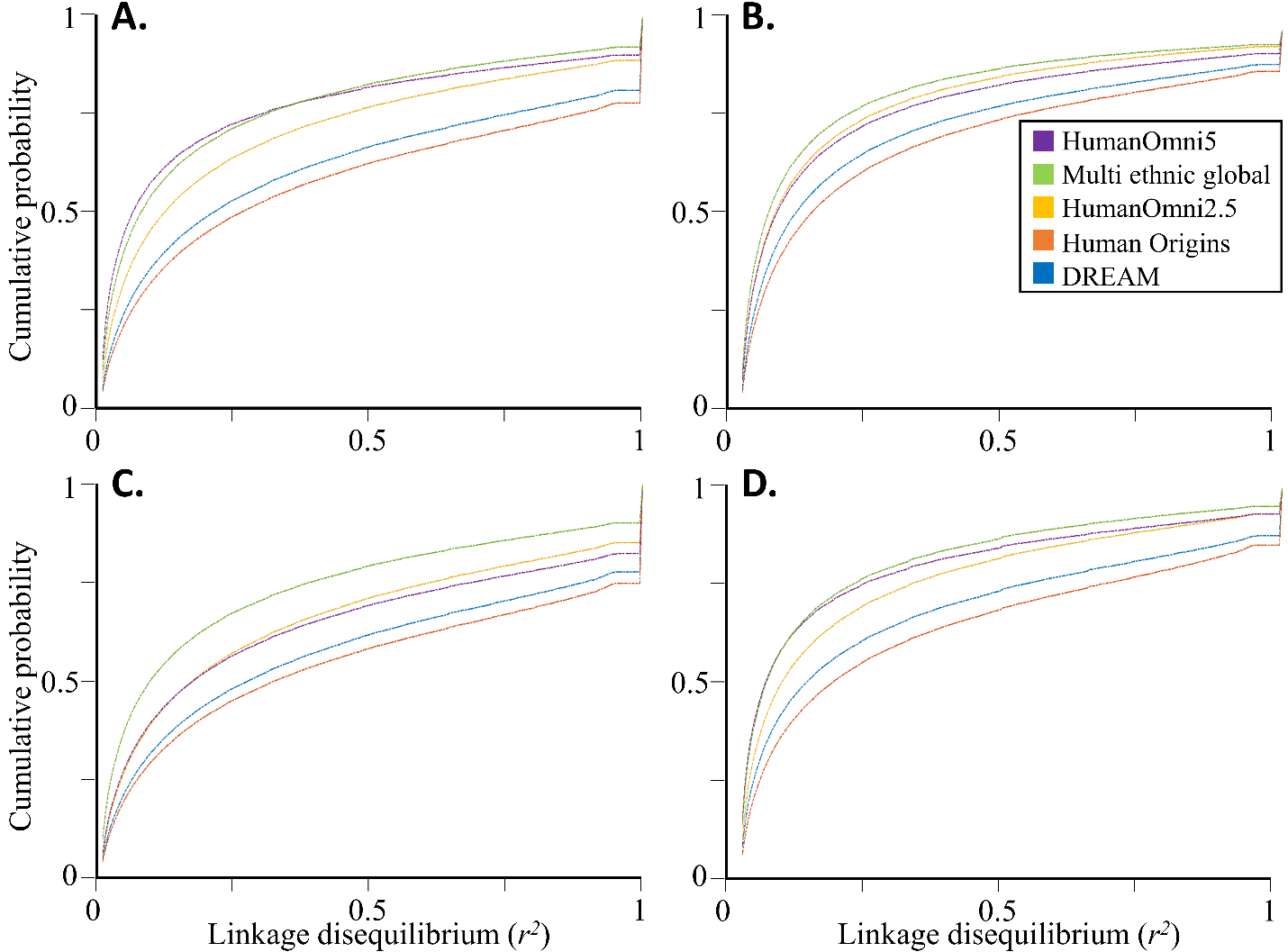


## Figure S8

Graph representation of Genographic individuals. Cluster colors correspond to the following geographical regions: North Europe (dark blue), South Europe (cyan), Near East (Middle East, Caucasus, and Pamiri – green), Indians (all except Northern Indians) (red), Oceania (North India and Oceanians) (pink), Africa (Africans and African Americans – magenta), and America (orange).

## Figure S9

Graph representation of Genographic individuals clustered into populations. Cluster colors correspond to the following geographical regions: North Europe (dark blue), South Europe (cyan), Near East (Middle East, Caucasus, and Pamiri – green), India (except Northern India) (red), Oceania (North India and Oceanians) (pink), Africa (Africans and African Americans – magenta), and America (orange). Node size was determined by the *Betweenness Centrality* measure, i.e., how often a node appears as an intermediate node between other pairs of nodes in the network.

## Figure S10

Population structure and CNV diversity. PCA of 80 1000 GP individuals plotted for biallelic deletions (A) and duplications (B) with colors representing regional populations. Individuals are projected along the first two PCs.


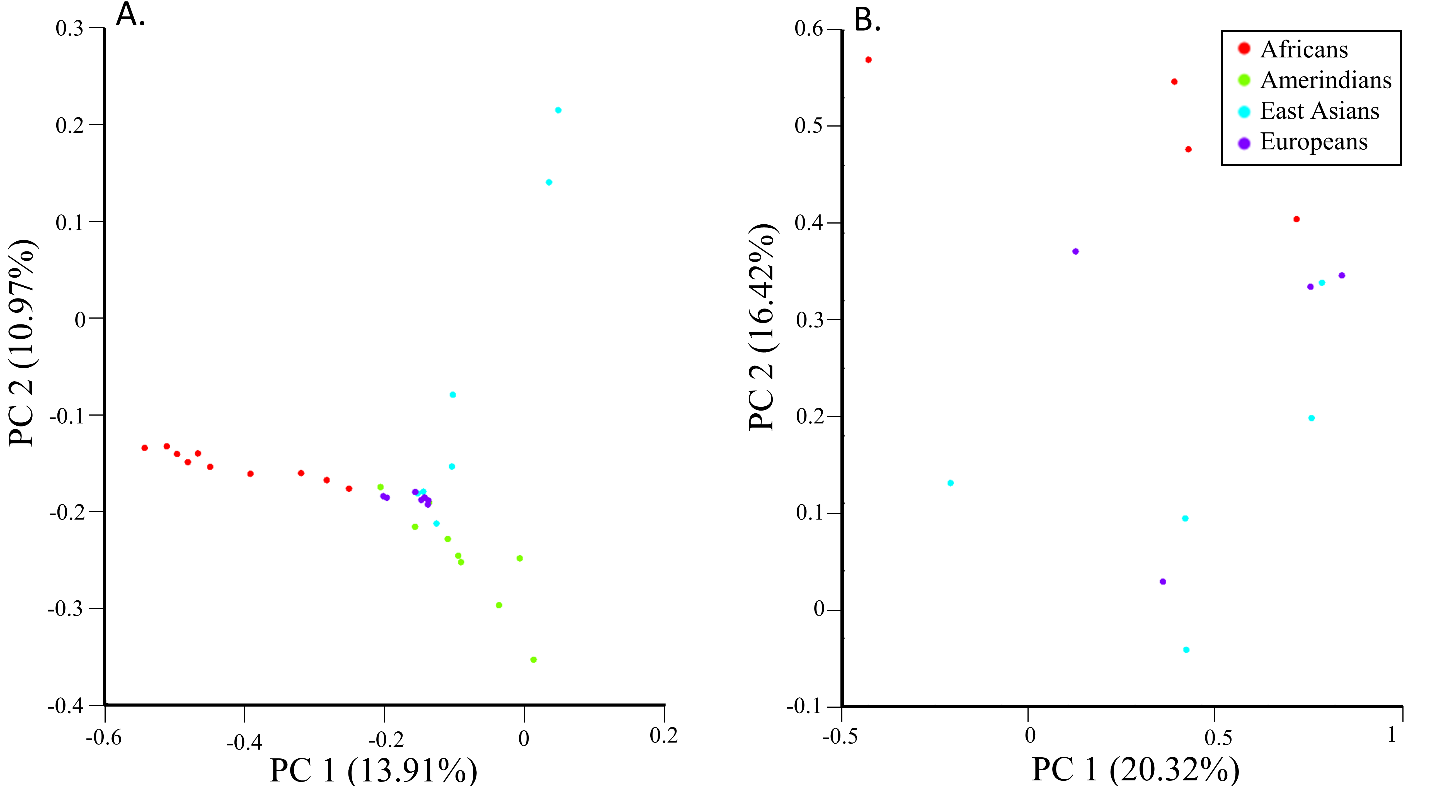


Supplementary table legend

## Table S1

Ancient DNA genomes used for DREAM’s design

## Table S2

Genes associated with adaptation that were included in DREAM.

## Table S3

Genes associated with forensic relevant traits that were included in DREAM.

## Table S4

Copy number variation (CNVs) included in DREAM.

## Table S5

The genes in included in DREAM

## Table S6

A comparison of the total coverage of IBD regions between the 1000 GP individuals, on the one hand, and Neanderthal and Denisovan, on the other hand, inferred using the 1000 GP and DREAM SNPs.

## Table S7

The admixture components and geographical coordinates of worldwide individuals assessed in GPS analysis.

## Table S8

The admixture components of the reference populations used in GPS analysis.

## Table S9

The geographical coordinates of the reference populations used in GPS analysis.

**REFERENCES**

Elhaik E, et al. 2013. The GenoChip: a new tool for genetic anthropology. Genome Biol. Evol. 5:1021-1031.

Elhaik E, et al. 2014. Geographic population structure analysis of worldwide human populations infers their biogeographical origins. Nat. Commun. 5.

International Society of Genetic Genealogy. 2015. Y-DNA SNP Index on Spreadsheet - 2015. <https://isogg.org/tree/ISOGG_YDNA_SNP_Index.html> (last accessed on September 30 2015).

mtDNA community. 2016. MtDNAPhylogeny. <http://www.mtdnacommunity.org/downloads/mtDNAPhylogeny.xml> (last accessed on March 15 2016).
