# Supplementary figures and images for: The Diversity of REcent and Ancient huMan (DREAM): A New Microarray for Genetic Anthropology and Genealogy, Forensics, and Personalized Medicine
Source: Genome Biol Evol. 2017 Nov 20;9(12):3225–37. doi: 10.1093/gbe/evx237 (PMC5726468; doi:10.1093/gbe/evx237)

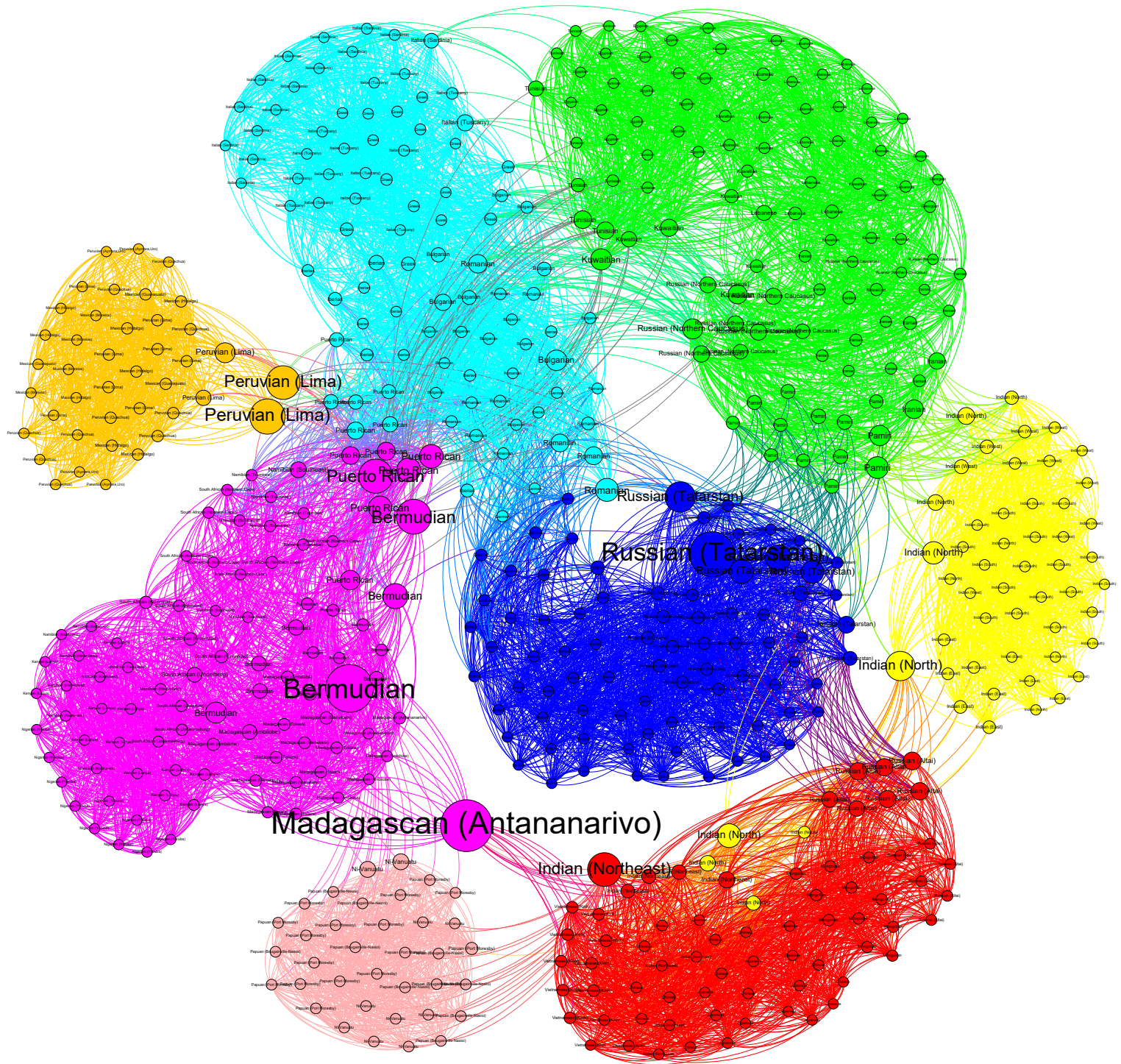

Supplement: Supplementary Figures and Tables [file evx237_supp.zip › Figure S8.pdf]

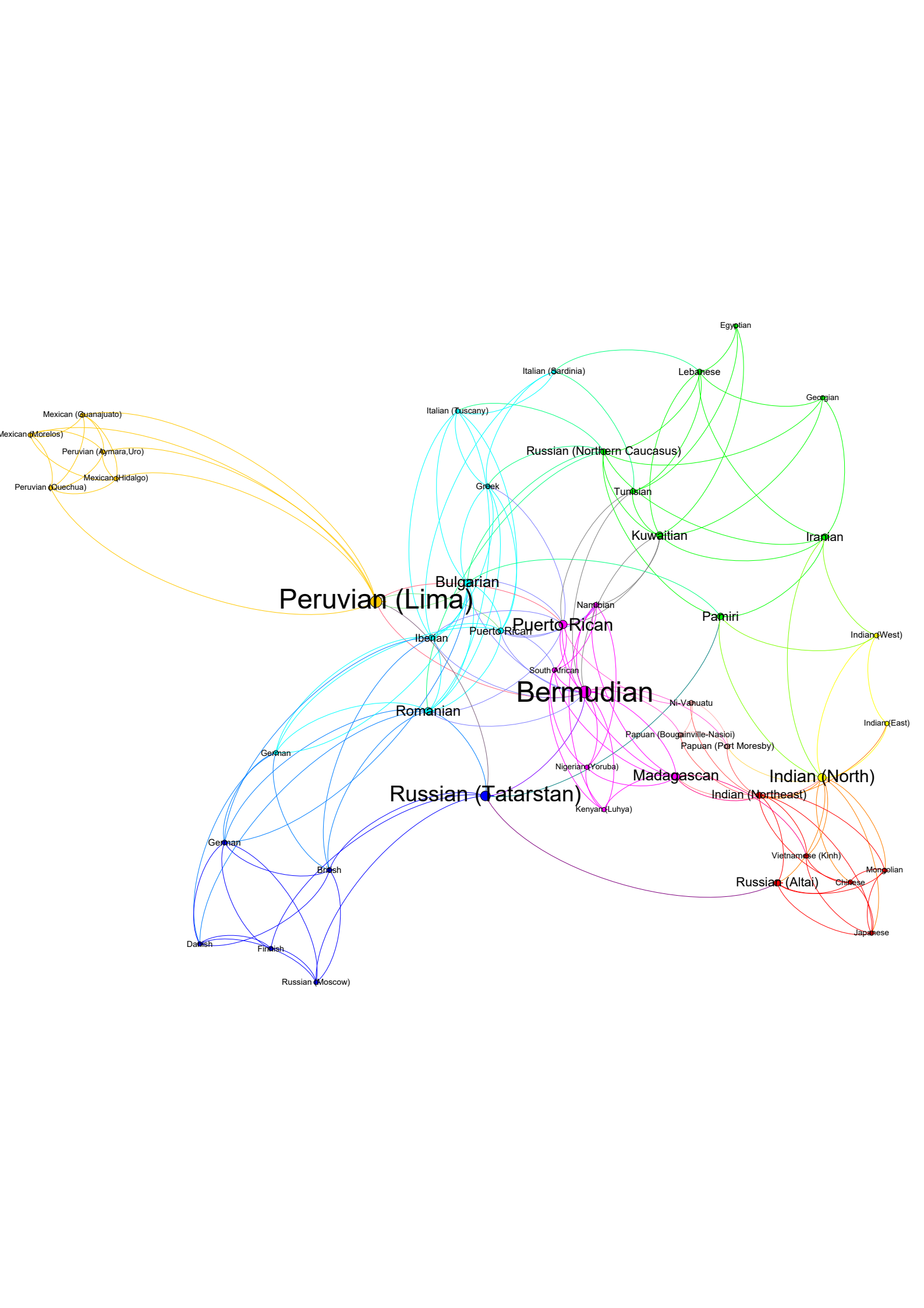

Supplement: Supplementary Figures and Tables [file evx237_supp.zip › Figure S9.pdf]
